# Factors associated with resistance of HIV-1 reservoir viruses to neutralization by autologous IgG antibodies

**DOI:** 10.1172/JCI194081

**Published:** 2025-07-29

**Authors:** Natalie F. McMyn, Joseph Varriale, Hanna W. S. Wu, Vivek Hariharan, Milica Moskovljevic, Toong Seng Tan, Jun Lai, Anushka Singhal, Kenneth Lynn, Karam Mounzer, Pablo Tebas, Luis J. Montaner, Rebecca Hoh, Xu G. Yu, Mathias Lichterfeld, Francesco R. Simonetti, Colin Kovacs, Steven G. Deeks, Janet M. Siliciano, Robert F. Siliciano

**Affiliations:** 1 Johns Hopkins University School of Medicine, Baltimore, Maryland, USA.; 2 Brigham and Women’s Hospital, Boston, Massachusetts, USA.; 3 Ragon Institute of MGH, MIT and Harvard, Cambridge, Massachusetts, USA.; 4 The Wistar Institute, Philadelphia, Pennsylvania, USA.; 5 University of Pennsylvania, Philadelphia, Pennsylvania, USA.; 6 Philadelphia Field Initiating Group for HIV-1 Trials, Philadelphia, Pennsylvania, USA.; 7 University of California San Francisco, San Francisco, California, USA.; 8 Maple Leaf Medical Clinic, Toronto, Ontario, Canada.; 9 Howard Hughes Medical Institute, Baltimore, Maryland, USA.

**Keywords:** AIDS/HIV, Infectious disease, Virology, AIDS vaccine, Adaptive immunity, Immunoglobulins

## Abstract

**BACKGROUND:**

Antiretroviral therapy (ART) prevents HIV-1 replication but does not eliminate the latent reservoir, the source of viral rebound if treatment is stopped. Autologous neutralizing antibodies (aNAbs) can block in vitro outgrowth of a subset of reservoir viruses and therefore potentially affect viral rebound upon ART interruption.

**METHODS:**

We investigated aNAbs in 31 people with HIV-1 (PWH) on ART.

**RESULTS:**

Participants fell into 2 groups based on a high or low fraction of aNAb-resistant reservoir isolates, with most isolates being aNAb-resistant (IC50 > 100 μg/mL). Time on uninterrupted ART was associated with higher aNAb resistance. However, pharmacodynamic analysis predicted that many isolates would be partially inhibited at physiologic IgG concentrations, to the same degree as by single antiretroviral drugs. Steep dose-response curve slopes, an indication of cooperativity, were observed for the rare isolates that were very strongly inhibited (> 5 logs) by aNAbs. Resistance to aNAbs was not fully explained by declining in aNAb titers and may be driven partially by ADCC-mediated elimination of infected cells carrying aNAb-sensitive viruses over long time intervals, leaving only aNAb-resistant viruses, which can contribute to viral rebound.

**CONCLUSION:**

Inhibition of reservoir viruses by aNAbs may be affected by dose-response curve slope, time on uninterrupted ART, waning of antibody responses, and selection against cells with aNAb-sensitive viruses.

**FUNDING:**

This work was supported by NIH Martin Delaney Collaboratories for HIV Cure Research grant awards UM1AI164556, UM1AI164570, and UM1AI164560, and the Howard Hughes Medical Institute

## Abstract 2

Most viruses in the latent HIV-1 reservoir are resistant to autologous neutralizing antibodies. This may reflect antibody-mediated immune selection and/or waning of antibody titers.

## Introduction

The major barrier to an HIV-1 cure is a population of latently infected resting CD4^+^ T cells that harbor replication-competent proviruses but are protected from immune clearance by viral latency (1–5). Antiretroviral therapy (ART) blocks new infection events but does not eliminate this latent reservoir (6–8). Upon ART interruption, viruses from the reservoir cause rebound viremia, typically within weeks (9–11). The reservoir decays slowly (t_1/2_ = 44 months) over the first 7 years of ART (12–14), but then increases slowly (t_2_ = 23 years) (15) due to infected cell proliferation (15–24) driven largely by antigen (25–29). In people with HIV-1 (PWH), particularly those on ART for more than 20 years, the reservoir is dominated by large clones of infected cells (15, 21–23). Importantly, autologous neutralizing antibodies (aNAbs) prevent rebound from a substantial but variable fraction of reservoir viruses, even those in large clones (30).

aNAbs target the HIV-1 envelope (Env) trimer on the virion surface and the plasma membrane of infected cells (31). Neutralization involves antibody binding to virions via Fab regions in a way that interferes with viral entry and thus blocks new infection events (32). Other antiviral activities of antibodies are mediated by Fc-dependent effector functions (33). HIV-1 has mechanisms to evade neutralization. Env is heavily glycosylated (34), and critical functional regions are shielded by 5 variable regions. Conserved antibody epitopes are rendered inaccessible through steric hindrance (35) and conformational masking (36). Another important mechanism is the rapid accumulation of mutations, which reflects the high error rate with which HIV-1 reverse transcriptase copies the viral genome in newly infected cells (37) and the high number of new infection events occurring daily during untreated infection (38, 39). Mutations accumulating in the 5 variable regions of the gp120 subunit of Env include point mutations, some of which alter the Env glycan shield (34), and insertions and deletions that range from 1 to several amino acids. The swift evolution of escape variants (40–42) enables HIV-1 to overcome circulating contemporaneous aNAbs as they arise.

Since aNAbs target variable regions and are thus largely strain specific, vaccine efforts have focused on broadly neutralizing antibodies (bNAbs) (43–46), which block entry of HIV-1 strains from different PWH (44, 45, 47). While aNAbs are induced within 2–3 months after infection in most PWH (41–43, 48), bNAbs only arise in a subset of PWH after extensive somatic hypermutation, which can take months to years (49–52). Administration of bNAbs can delay viral rebound after ART interruption (53–57). However, viremia typically rebounds when bNAb concentrations fall to subinhibitory levels. bNAbs can also target infected cells through antibody-dependent cellular cytotoxicity (ADCC) (58–60). Most bNAb trials in PWH have not produced reservoir reduction, likely because latently infected cells do not express viral proteins (57, 61, 62). However, in one trial, a 46% reduction was measured by Q4PCR (55).

Recent studies suggest that selection can operate on reservoir cells. Cells carrying intact proviruses decay faster than cells carrying defective proviruses during the first years of ART, possibly reflecting immune mechanisms targeting cells with transcriptionally active, intact proviruses (63, 64). Similarly, while proviruses are typically integrated within active genes (65, 66), an increased fraction of intact proviruses in intergenic regions and chromosomal locations repressive for transcription is reported in PWH initiating ART early (67), PWH on long-term ART (68), and elite controllers (69). Reservoir selection through innate immune mechanisms, possibly involving NK cells, has also been reported (70–72). Thus, antibodies involved in ADCC may play a role in changing reservoir composition over time. Furthermore, immune pressure from aNAbs has been seen in multiple studies. Viruses rebounding after treatment interruption were more resistant to aNAbs, possibly reflecting aNAb-mediated inhibition of outgrowth of sensitive viruses (30, 73, 74). Together, these findings indicate that, over time, reservoir composition may be altered by both the clonal expansion of infected cells and selection against cells expressing viral antigens.

Given that rebound from some reservoir viruses is blocked by aNAbs (30), we examined the neutralizing activity of contemporaneous autologous IgG antibodies against inducible, replication-competent HIV-1 isolates from a large cohort of PWH on ART, including many who had had stable suppression of viremia for 20–25 years. We defined the extent and durability of aNAb responses. We also investigated antibody-based selection against cells carrying aNAb-sensitive viruses.

## Results

### Study participants.

We studied 31 PWH, including 9 participants from our initial aNAb study (30) who had extensive reservoir sequencing. The mean time on combination ART was 15.9 years (range 4.5–26.7 years, Supplemental Table 1; supplemental material available online with this article; https://doi.org/10.1172/JCI194081DS1). The demographics were 87.1% male, 12.9% female, 64.5% Black, 32.3%, White, and 3.2% Pacific Islander. Participants had a mean CD4 nadir of 225 cells/μL and a mean age of 54 years at the last sample collection. Viral outgrowth results for 25 participants were previously reported in Bertagnolli et al. (30) and McMyn et al. (15). Those studies showed that replication-competent virus could be readily isolated from study participants, even those on ART for more than 20 years, at frequencies between 0.05–16.25 infectious units per million (IUPM) resting CD4^+^ T cells, and that aNAbs could suppress outgrowth of a subset of these viruses.

All participants initiated ART during chronic infection. Longitudinal plasma HIV-1 RNA levels and CD4 counts are in Supplemental Figure 1 and McMyn et al. (15). Five participants had short periods of ART interruption. Three of these (DEL-SPC-015, -017, and -019) were in ACTG clinical trial A5340 (IDs A02, A06, and A13, respectively) which involved administration of the bNAb VRC01 and an analytic treatment interruption (ATI). Plasma HIV-1 RNA levels during the ATI were previously reported (54). DEL-SPC-012 and JH448 had treatment interruptions due to nonadherence. All participants had plasma HIV-1 RNA levels below the detection limit (< 20–40 copies/mL) at the time of sampling. Small, isolated blips, transient increases in HIV-RNA above the detection limit (75, 76), were not considered in calculating time on uninterrupted ART.

### Sensitivity of reservoir isolates to aNAbs.

To determine the aNAb sensitivity of replication-competent proviruses from a diverse group of PWH, we performed quantitative viral outgrowth assays (QVOAs) (77, 78) using resting CD4^+^ T cells. Cultures from 28 of 31 participants had greater than or equal to 5 p24^+^ outgrowth wells, for a total of 591 independent isolates. Full length *env* sequencing revealed 138 different (distinct) *env* sequences. The other 453 sequences were identical to other sequences from the same participant. Among sequences found more than once, there were 69 sets of identical sequences ranging in size from 2 to 59 isolates. Distinct *env* sequences were cloned into expression vectors for pseudovirus generation and neutralization assays as previously described (30). Because nonspecific inhibition can be observed at IgG concentrations over 100 μg/mL, we arbitrarily designated isolates as resistant if the IC_50_ for autologous IgG was over 100 μg/mL.

**Figure 1:**
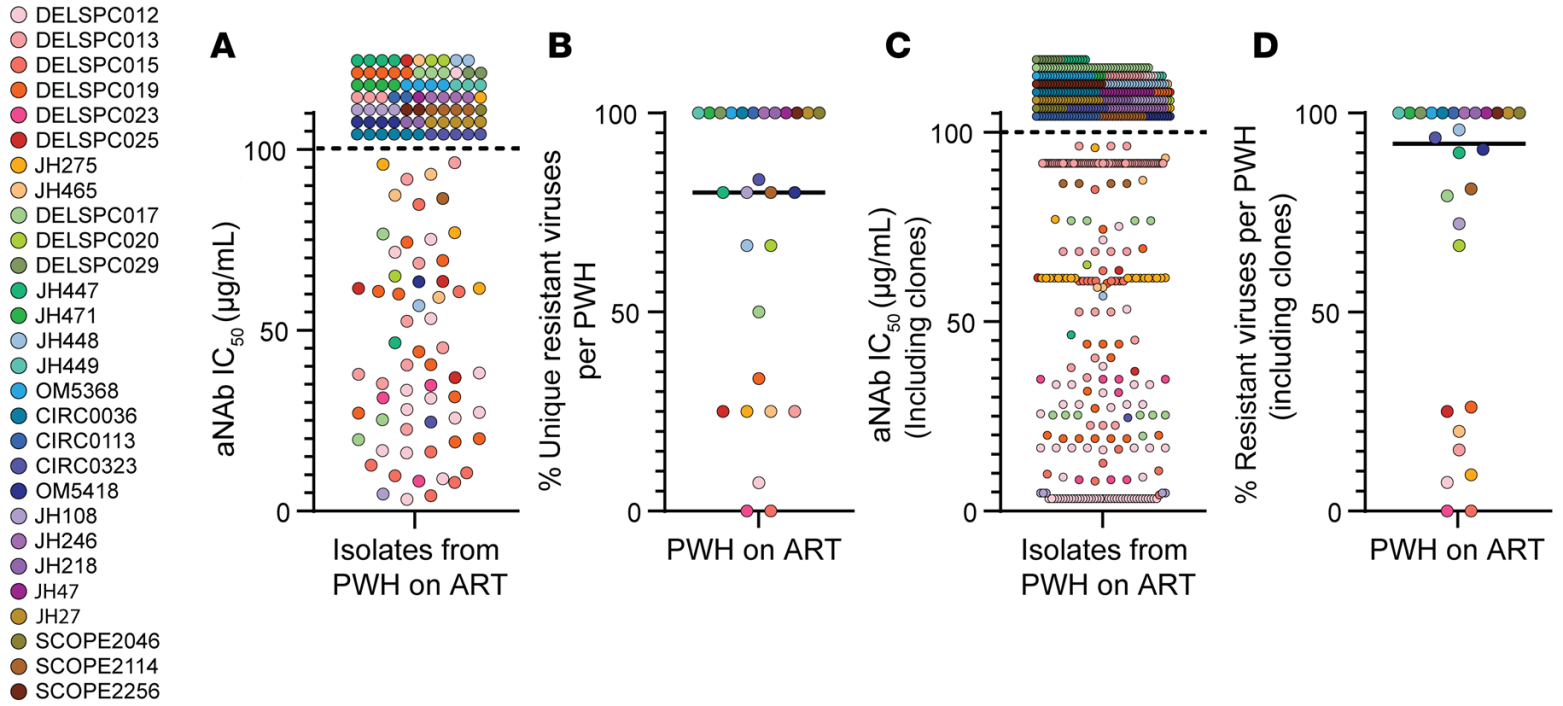
Variation in sensitivity of reservoir isolates to aNAbs. (**A**) aNAb IC_50_ values were determined in TZM.bl-based neutralization assays for distinct pseudoviruses generated from outgrowth viruses from PWH on ART (*n* = 138 isolates). Autologous IgG concentrations up to 100 μg/mL were used. Circles represent distinct isolates, and colors represent participants. (**B**) Percentage of distinct outgrowth viruses resistant (IC_50_ > 100 μg/mL) to neutralization by contemporaneous aNAbs per PWH. Each data point (*n* = 28) represents the % of resistant viruses among the total number of viruses tested in each PWH. Bar represents median. (**C**) aNAb neutralization of distinct isolates shown in **A** with additional data points representing independent isolates from the same PWH with *env* sequences identical to those shown in **A**, *n* = 591. IC_50_ values determined for one member of a set of isolates with identical *env* sequence were used for all members of the set. (**D**) Percentage of outgrowth viruses resistant to neutralization (IC_50_ > 100 μg/mL) by aNAbs per PWH, *n* = 28, using values from (**C**). All isolates from each participant, including sequence-identical isolates, are considered in the percentage calculation. Bar represents median.

Of 138 distinct HIV-1 *env* pseudoviruses from 28 PWH 55% (76/138) were resistant to neutralization (Figure 1A). Since some individuals had fewer distinct isolates to test due to high reservoir clonality and/or smaller reservoir size, we determined the fraction of resistant isolates per PWH to normalize for different numbers of isolates per participant (Figure 1B). There was wide variation in the fraction of resistant sequences (0%–100%, median 80%).

### Effects of reservoir clonality.

The analyses presented above consider only distinct variants. However, reservoir composition is also influenced by clonal expansion. Therefore, we analyzed resistance to aNAbs considering all isolates from each donor, not just distinct sequences. Of 591 total isolates, 60% (357/591) were resistant to contemporaneous aNAbs (IC_50_ > 100 μg/mL, Figure 1C). After normalizing for different numbers of isolates for each participant, we found a median of 92% resistant viruses per PWH, again with very high person-to-person variation (0%–100%) (Figure 1D). For 43% of participants, all reservoir isolates were resistant (Figure 1, B and D).

### Factors associated with aNAb resistance.

**Figure 2:**
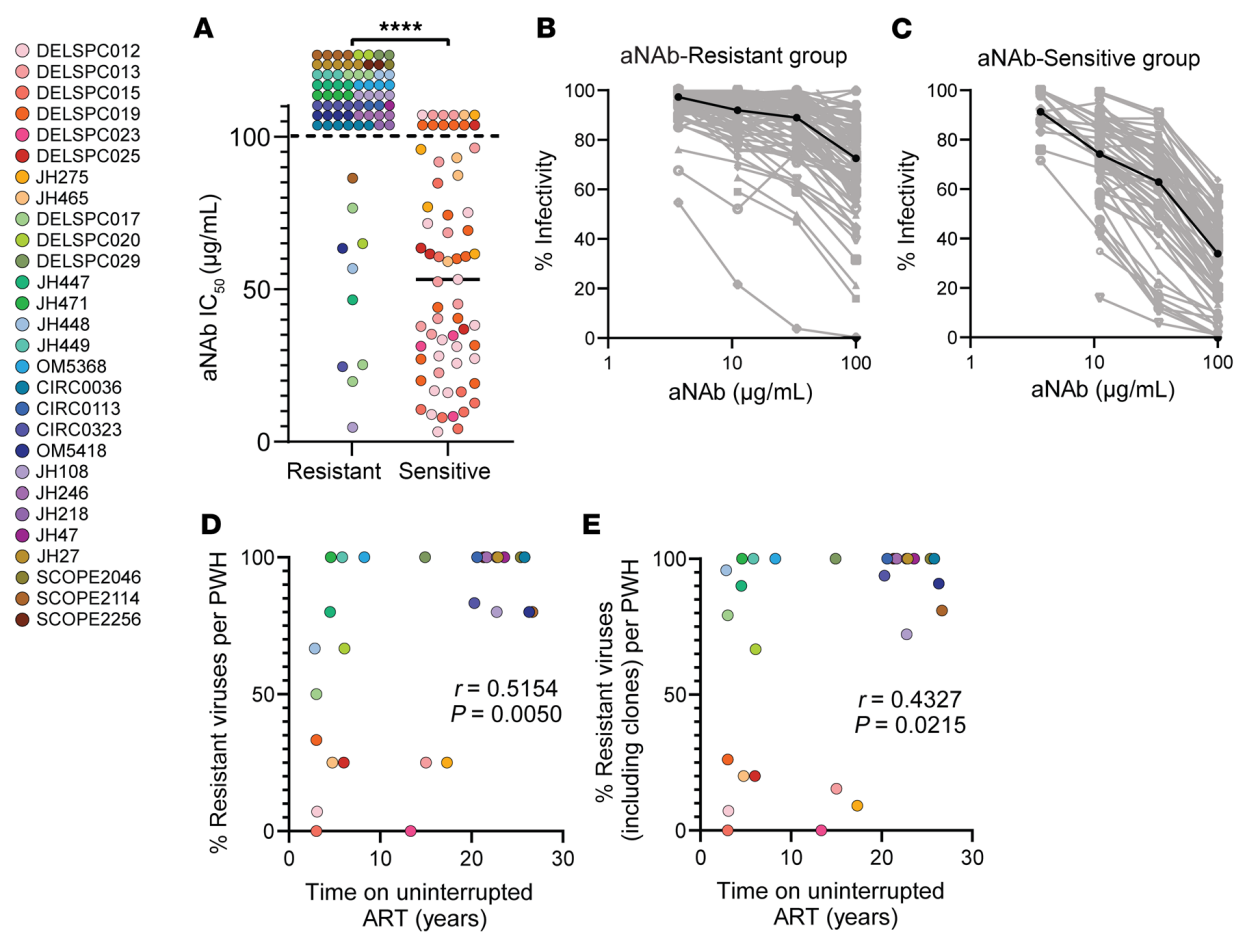
Understanding individual variation in aNAb resistance. PWH on ART were divided into 2 groups, those with aNAb-resistant or aNAb-sensitive reservoirs, based on Figure 1D. The aNAb-resistant group had greater than or equal to 67% resistant isolates while the aNAb-sensitive group had less than or equal to 26% resistant isolates. (**A**) aNAb IC_50_ values for distinct pseudoviruses for the aNAb-resistant (*n* = 74 isolates) and aNAb-sensitive groups (*n* = 64 isolates). Circles represent distinct isolates, and colors represent participants. The Mann-Whitney test was used to calculate significance. ****P < 0.0001. Bar represents median. (**B** and **C**) Dose-response curves for the inhibition of pseudovirus infectivity by autologous IgG for isolates from the aNAb-resistant (**B**, *n* = 74 isolates) and aNAb-sensitive (**C**, *n* = 64 isolates) groups. The median curve is overlayed in black. Data are reported as a mean % of maximum infectivity from triplicate measurements. (**D**) Percentage of resistant viruses per PWH from Figure 1B correlates with time on uninterrupted ART (Spearman’s correlation, *n* = 28). (**E**) Percentage of resistant viruses per PWH, including sets of identical sequences from Figure 1D, correlates with time on uninterrupted ART (Spearman’s correlation, *n* = 28).

Figure 1D reveals a separation in aNAb resistance between 20 participants with high resistance (67%–100% resistant isolates per PWH) and 8 participants with high sensitivity (0%–26% resistant isolates per PWH). Of 74 distinct viral isolates from the high resistance group, 64 isolates (86%) were neutralization resistant (Figure 2A). This fraction was significantly higher than the 19% observed in the group with high aNAb sensitivity (12/64 distinct aNAb-resistant isolates, *P* < 0.0001, Figure 2A). The difference was visually apparent in flatter neutralization curves for the aNAb-resistant group (Figure 2, B and C). The impact of dose-response curve slope is described below. For both groups, resistant variants were found among sets of isolates with identical *env* sequences as well as sequences observed only once (Supplemental Figures 2 and 3). There were no correlations between aNAb IC_50_ values and the number of identical sequences in a set for either group (Supplemental Figure 4).

We next explored other explanations for differences in aNAb resistance. No significant differences between the 2 groups were found for CD4 nadir, a proxy for time of untreated infection (Supplemental Figure 5A), or reservoir size, based on QVOA measurements (Supplemental Figure 5B). We then evaluated differences in total time on ART. Although the mean time on ART for the aNAb-resistant group was higher (16.9 vs 10.9 years), the difference was not significant (*P* = 0.1018, Supplemental Figure 5C). This time included the time that 5 participants (DEL-SPC-012, -015, -017, -019, and JH448) experienced treatment interruptions. Therefore, we compared time on uninterrupted ART or time since last period of measurable viremia. This analysis resulted in significant differences. For the aNAb-resistant group, the average time of uninterrupted ART was 16.5 years, significantly higher than the average time of 8.2 years for the aNAb-sensitive group (*P* = 0.0277, Supplemental Figure 5D). These results suggest that ART interruptions and shorter times on uninterrupted ART may affect neutralization sensitivity.

To further investigate the relationship between aNAb resistance and time on ART, we analyzed the correlation between aNAb IC_50_ values or % resistance per person and time on ART or time on uninterrupted ART. There was a significant positive correlation between the aNAb IC_50_ values of distinct isolates from both the aNAb-sensitive and aNAb-resistant groups and time on ART (Spearman *r* = 0.2058, *P* = < 0.0155; Supplemental Figure 6A). We found a more significant positive correlation between aNAb IC_50_ values of distinct isolates and time on uninterrupted ART (Spearman *r* = 0.4644, *P* = < 0.0001, Supplemental Figure 6B). When the analysis was expanded to include all independent isolates from each PWH and not simply the distinct isolates, the correlation was highly significant for both time on ART (Spearman *r* = 0.2441, *P* = < 0.0001) and time on uninterrupted ART (Spearman *r* = 0.3909, *P* = < 0.0001). Because these analyses are affected by the number of isolates per donor, we also examined the correlation between the fraction of distinct isolates that were resistant in each PWH and the time on uninterrupted ART. We found a significant positive correlation (Spearman *r* = 0.5154, *P* = 0.0050; Figure 2D). When the analysis included all isolates from each PWH, including identical sequences, there was a weaker positive correlation (Spearman *r* = 0.4327, *P* = 0.0215, Figure 2E). When the total time on ART was used, no significant correlation was found. Although conclusions depended on how time on ART was defined, longer times on ART were generally associated with greater aNAb resistance, perhaps indicating gradual selection against cells carrying aNAb-sensitive viruses (see below).

To determine whether the observed aNAb resistance of replication-competent isolates was representative of other persistent proviruses not detected in outgrowth assays, we assessed aNAb sensitivity of intact *env* sequences amplified from resting CD4^+^ T cells from 6 PWH on long-term ART (> 21 years) (15). The high aNAb resistance of outgrowth viruses was also observed for these proviruses (Supplemental Figure 2). Across the 6 participants, 16 of 17 distinct proviruses were resistant. The 1 sensitive isolate had an IC_50_ of 96.12 μg/mL. These results provide evidence for a latent reservoir dominated by resistant variants regardless of inducibility. The finding that most reservoir viruses are resistant to neutralization by contemporaneous IgG, especially in PWH on long-term ART, suggests that, over long time intervals, there may be a selection against cells carrying aNAb-sensitive viruses. Alternatively, a decline in aNAb concentration may explain reduced neutralization (see below).

### aNAb resistance at physiologic IgG concentrations.

**Figure 3:**
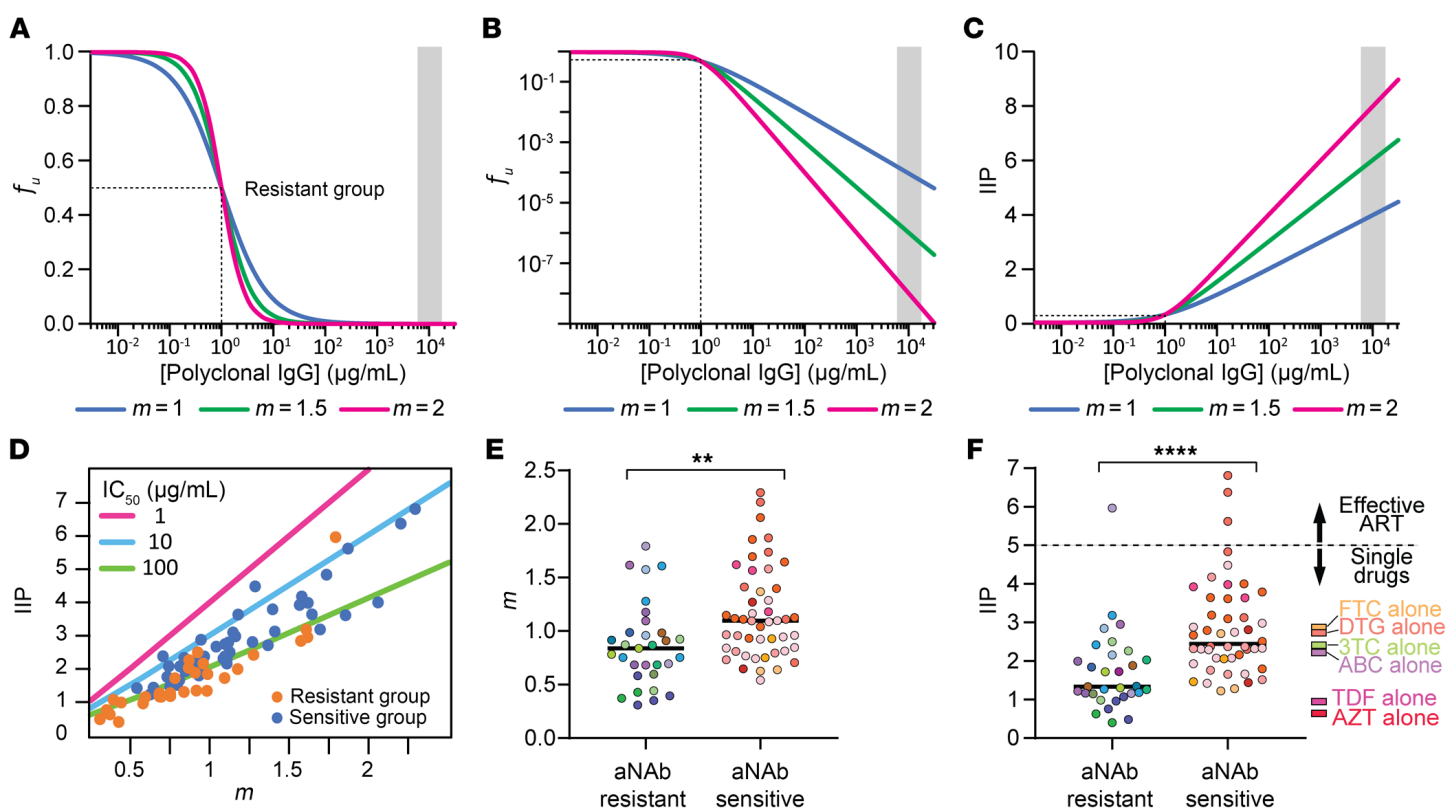
Inhibitory effect of aNAbs at physiologic IgG concentrations. (**A**) Hypothetical dose-response curves for inhibition of infection by autologous IgG antibodies. The fraction of infection events not inhibited (fraction unaffected or *f_u_*) declines with increasing antibody concentrations. Curves show inhibition by 3 different antibody preparations that have the same IC_50_ (1 μg/mL, dotted line) but different slopes (*m*), a measure of cooperativity. In vitro neutralization assays are conducted at IgG concentrations well below physiologic IgG concentrations (shaded area). (**B**) Curves from **A** plotted with a logarithmic *y*-axis, illustrating the dramatic effect of *m* on inhibition. (**C**) Instantaneous inhibitory potential (IIP), the logs of inhibition of a single round of infection, for the same antibody preparations. (**D**) Effect of *m* on IIP for distinct isolates from the aNAb-resistant (orange, *n* = 31) and aNAb-sensitive (blue, *n* = 48) groups. IC_50_ value trend lines are shown at 3 indicated concentrations. For many resistant isolates, *m*, IC_50_, and IIP, could not be determined because infection did not consistently decrease with increasing IgG concentrations. (**E**) Distribution of slope values for the isolates from **D**. Circles represent distinct isolates, and colors represent participants. Bar represents median. Significance was calculated using the Mann Whitney test. **P < 0.01. (**F**) Distribution of IIP values for the isolates from **D**. Dotted line indicates the IIP value above which effective suppression of viral replication by combination ART regimens is observed (85). IIP values for single antiretroviral drugs at average plasma concentrations are shown (80, 85). Significance was calculated using the Mann Whitney test. ****P < 0.0001.

The above results indicate that in many PWH on ART, most replication-competent reservoir viruses are not strongly neutralized by contemporaneous autologous IgG in in vitro assays. However, these assays are carried out using polyclonal IgG concentrations in the μg/mL range, while the in vivo plasma concentration of IgG is 7–16 mg/mL (79). From conventional dose-response curves, it is difficult to ascertain the degree of inhibition at in vivo concentrations (Figure 3A). Therefore, we used previously described pharmacodynamic metrics to predict the in vivo effects of aNAbs (80–82). The dose response curve can be described using the median effect equation (83):


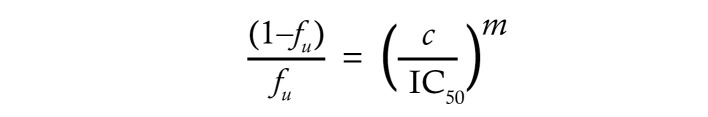


 Equation 1

where ƒ_u_ is the fraction of infection events unaffected (not inhibited) by antibodies at concentration (*c*) given the IC_50_ and the dose response curve slope (*m*) or Hill coefficient, a measure of cooperativity that is equal to 1 for noncooperative processes. Slopes greater than 1 give steeper dose-response curves, but differences in inhibition at physiologic IgG concentrations are only apparent if the y-axis values are displayed on a log scale (Figure 3B). The inverse of this plot gives the instantaneous inhibitory potential (IIP),


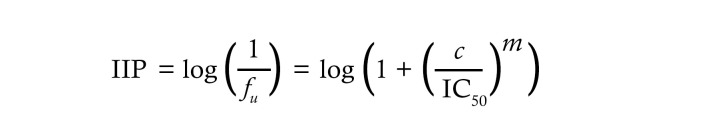


 Equation 2

an intuitive metric of antiviral activity, which is the number of logs by which single round infection events are reduced at a given antibody concentration (Figure 3C) (80–82). IIP depends on both IC_50_ and *m* (Figure 3D). Because of their exponential relationship, IIP is strongly influenced by *m*. Figure 3D shows calculated IIP at 10 mg/mL of IgG based on experimental measurements of IC_50_ and *m* for distinct isolates from the aNAb-resistant and aNAb-sensitive groups. As expected, IIP values are strongly influenced by *m* (linear regression R^2^ = 0.77 and 0.83 for isolates from the resistant and sensitive groups, respectively). Figure 3E shows slope values for the 2 groups. Although antigenic and antibody heterogeneity can reduce slope, we observed slopes greater than 1, indicative of positive cooperativity, as is seen for HIV-1 protease inhibitors, nonnucleoside reverse transcriptase inhibitors, entry inhibitors, and some bNAbs (80–82, 84).

This analysis allows extrapolation of dose-response curves to physiologic IgG concentrations (Figure 3F). For the aNAb-resistant group, most isolates were weakly inhibited with a median IIP of 1.3, just above the level of nonspecific inhibition observed with control IgG. For the aNAb-sensitive group, many isolates were inhibited with a median IIP of 2.4, meaning greater than 2 logs of inhibition at in vivo IgG concentrations, comparable to single antiretroviral drugs (80–82). Effective combination ART regimens produce greater than 5 logs of inhibition (85). Only 4 isolates were inhibited by aNAbs with IIP greater than 5 (Figure 3F). These isolates had IC_50_ values less than 13 μg/mL and slopes greater than 1.7 (Supplemental Table 2). This analysis excluded 43 distinct isolates from the aNAb-resistant group and 16 from the aNAb-sensitive group because slopes and IIP values could not be calculated due to poor inhibition (nondecreasing dose response curves, maximum inhibition < 20%, or median effect plot R^2^ < 0.8). Together, these results indicate that, like single antiretroviral drugs, aNAbs can reduce replication of many reservoir viruses but not sufficiently enough to prevent selection of resistant variants.

### Sensitivity of reservoir viruses to neutralization by bNAbs.

**Figure 4:**
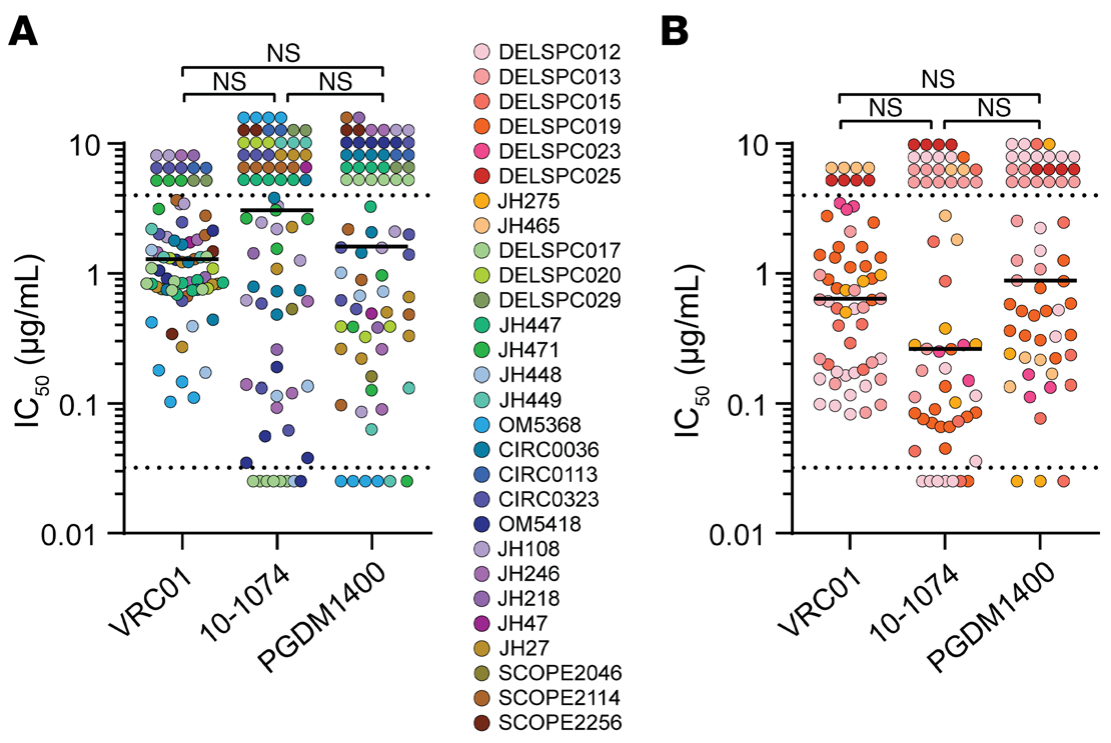
Sensitivity of outgrowth viruses to bNAbs. (**A**) TZMbl-based neutralization assays were conducted with VRC01, 10-1074, and PGDM1400 and pseudoviruses generated from outgrowth viruses (*n* = 74) from the aNAb-resistant group. (**B**) Neutralization assays with pseudoviruses generated from outgrowth viruses (*n* = 58) from the aNAb-sensitive group. IC_50_ values above the highest antibody concentration tested are shown above the dotted lines at 4 μg/mL. Values below the lowest concentration tested are shown below the dotted lines at 0.032 μg/mL. Circles represent distinct isolates, and colors represent participants. We used a nonparametic 1-way ANOVA (Friedman test) followed by Dunn’s test for multiple comparisons to calculate significance. Bar represents median.

In light of the high proportion of aNAb-resistance after long-term ART, we explored whether these viruses were resistant to 3 clinically relevant bNAbs: VRC01, 10-1074, and PGDM1400, which bind to the CD4 binding site, the V3 glycan site, and the V2 apex, respectively (45). Many isolates from the aNAb-resistant group were neutralized by bNAbs (median bNAb IC_50_: 1.280, 2.857, and 1.595 μg/mL for VRC01, 10-1074, and PGDM1400, respectively; Figure 4A). For 90% of donors in the aNAb-resistant group, all isolates were sensitive to at least one bNAb with IC_50_ values less than 4 μg/mL, and 55% of donors had isolates sensitive to at least 2 bNAbs (Supplemental Table 3). Thus, although viral isolates from these PWH were aNAb resistant, many isolates were sensitive to neutralization by 1 or more bNAbs. Only 4 isolates from 2 PWH were resistant to all 3 bNAbs. Importantly, bNAb sensitivity was not a predictor of aNAb sensitivity. The 10 isolates with aNAb IC_50_ values less than 100 μg/mL had similar bNAb sensitivities to isolates with aNAb IC_50_ values greater than 100 μg/mL (Supplemental Table 3). Ultimately, the majority of viruses in the aNAb-resistant group could be effectively neutralized by some bNAbs, ruling out general neutralization resistance as an explanation for high aNAb resistance in that group. Similar results were obtained for the aNAb sensitive group (Figure 4B).

### Some aNAb responses persist over long times on ART.

**Figure 5:**
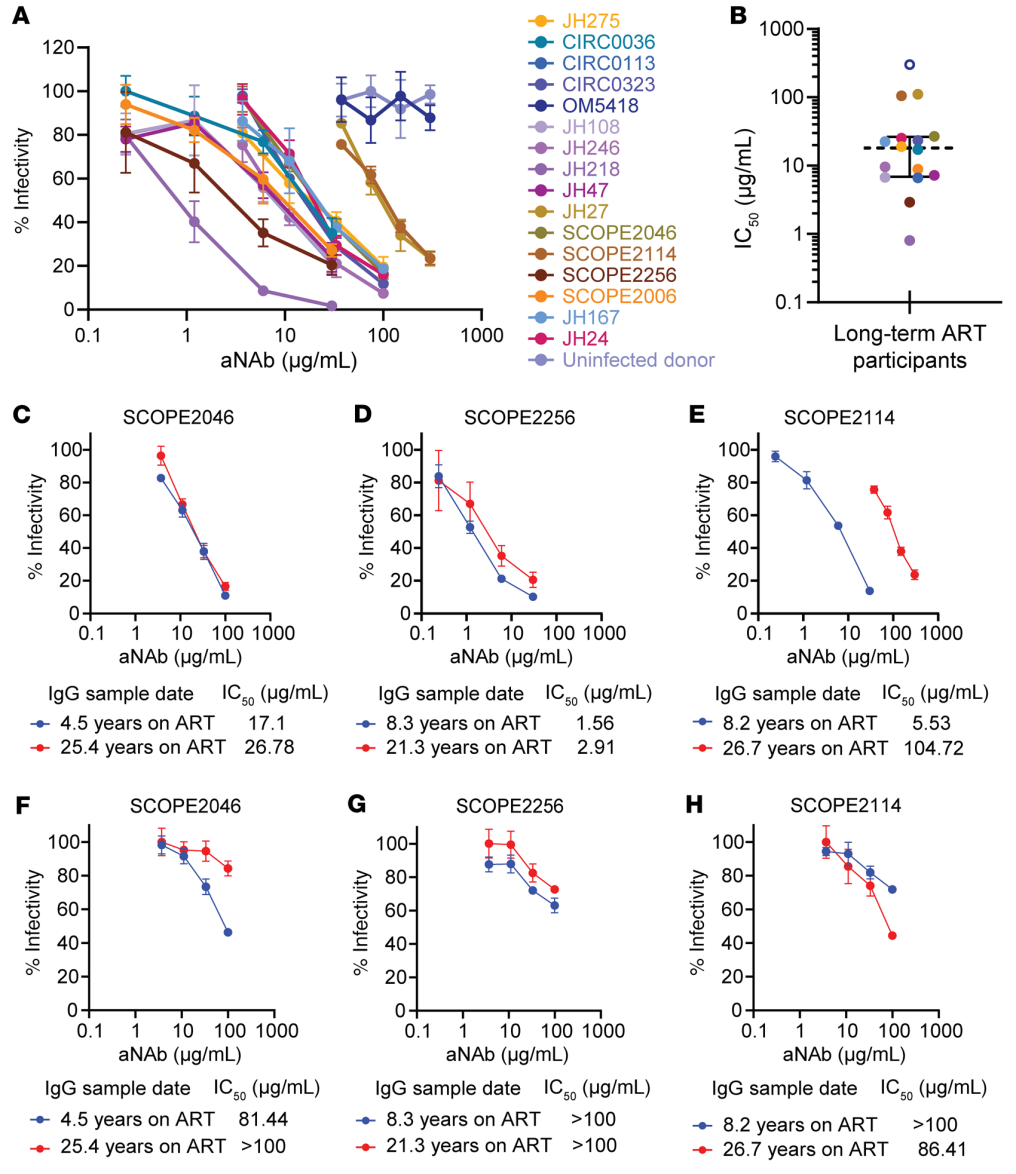
Persistence of neutralizing antibodies to HIV-1 Env in PWH on long-term ART. (**A**) Dose-response curves for inhibition of SF162 pseudovirus infection of TZM.bl cells by IgG purified from 16 PWH on long-term ART and 1 uninfected donor. (**B**) IC_50_ values from dose-response curves in **A**. A value above the highest concentration tested (300 μg/mL) is shown in an open symbol. Dashed line represents median, and solid bars represent interquartile range. *n* = 16. (**C**) Dose-response curves for the inhibition of SF162 pseudovirus infection of TZM.bl by early and 20-year IgG samples from SCOPE2046, (**D**) SCOPE2256, and (**E**) SCOPE2114. (**F**) Dose-response curves for the inhibition of TZM.bl infectivity of one autologous outgrowth virus by early and 20-year autologous IgG collection timepoints from SCOPE2046, (**G**) SCOPE2256, and (**H**) SCOPE2114. Data for (**A** and **C**–**H**) are reported as percentage (mean, SEM) of infectivity from triplicate measurements.

Another explanation for the large proportion of outgrowth viruses resistant to aNAbs is that, without antigen, the anti-HIV antibody response wanes. Therefore, we studied antibody responses in participants on very long-term ART. Western blot analysis of IgG from a set of participants who had been on ART for an average of 22.6 years showed that some anti-HIV antibodies persisted despite prolonged suppression of viremia (Supplemental Figure 7). To examine whether neutralizing antibody responses persist, we first tested neutralization of pseudoviruses carrying the *env* gene of HIV-1 SF162, a tier 1 virus that is highly sensitive to neutralization by antibodies from many PWH (86, 87). Neutralization assays with SF162 pseudovirus were performed with IgG from 16 participants who had been on very long-term ART (mean 22.8 years), 12 of whom were from the aNAb-resistant group. SF162 pseudovirus was neutralized by IgG from 15 of 16 participants (Figure 5, A and B). The interquartile range of IC_50_ values was 6.9–26.4 μg/mL, with a median of 18.1 μg/mL (Figure 5B). No neutralizing activity was detected with IgG from an HIV-seronegative donor. For 3 participants with only 1 or no QVOA outgrowth viruses (SCOPE2006, JH167, JH24), participant IgG neutralized SF162 pseudovirus with IC_50_ values less than 26 μg/mL. This suggests that a smaller reservoir did not result in reduced antibody stability over time. Together, these results demonstrate that some neutralizing antibodies to the HIV-1 Env protein are detectable in most PWH even after more than 20 years of treatment, regardless of reservoir size.

Antibodies that neutralize SF162 recognize an epitope that is conserved and immunogenic. Thus, these antibodies are distinct from the aNAbs, which generally show limited heterologous neutralization (30). Examining the stability of aNAb responses in PWH on long-term ART is difficult because longitudinal plasma samples spanning 2 decades are rarely available. To determine if aNAb titers persist over long time intervals, we identified 3 study participants in the aNAb-resistant group who had been on ART for over 20 years and had a plasma sample from more than a decade earlier. First, we compared aNAb neutralization of SF162 between the early and 20-year IgG samples. For 2 participants, neutralizing activity remained similar, while in 1 participant there was a more than 1 log increase in IC_50_ (Figure 5, C–E).

We then compared neutralizing activity between the early and 20-year IgG preparations against autologous outgrowth viruses from the 20-year timepoint (Figure 5, F–H). For participant SCOPE2046, 11 of 11 isolates had identical *env* sequences, potentially reflecting an expanded clone (Supplemental Figure 2). For this Env, the aNAb IC_50_ increased from 81.44 to over 100 μg/mL over 20.9 years of ART, demonstrating some waning of the aNAb response (Figure 5F). Interestingly, neutralization of SF162 pseudovirus remained relatively stable over the same period (Figure 5C). For the second participant (SCOPE2256), 23 of 26 isolates had identical *env* sequences (Supplemental Figure 2). For this Env, modest neutralizing activity persisted over 13 years of ART (Figure 5G) as did neutralizing activity against SF162 (Figure 5D). The other 2 outgrowth isolates from SCOPE2256 were identical and remained resistant (IC_50_ > 100 μg/mL) at both timepoints (not shown). For the third participant (SCOPE2114), 1 of 5 distinct outgrowth viruses was neutralized by IgG from the late time point (IC_50_ = 86.41 μg/mL), while IgG from 18.5 years earlier had less neutralizing activity (IC_50_ > 100 μg/mL) (Figure 5H). An opposite trend was observed for neutralization of SF162 pseudovirus (Figure 5E). The other 4 outgrowth viruses from SCOPE2114 remained resistant (IC_50_ > 100 μg/mL) at both timepoints (not shown). Together, these observations demonstrate that aNAb responses can persist, improve, or wane, depending on the individual and the viral sequence tested. The waning of aNAb responses might explain the observed neutralization resistance in some PWH on ART, but in cases where the aNAb response remains stable, it is possible that the relevant antibodies eliminate reservoir cells carrying sensitive variants, leaving only aNAb-resistant viruses.

### Autologous IgG antibodies can mediate ADCC against cells infected with reservoir viruses.

**Figure 6:**
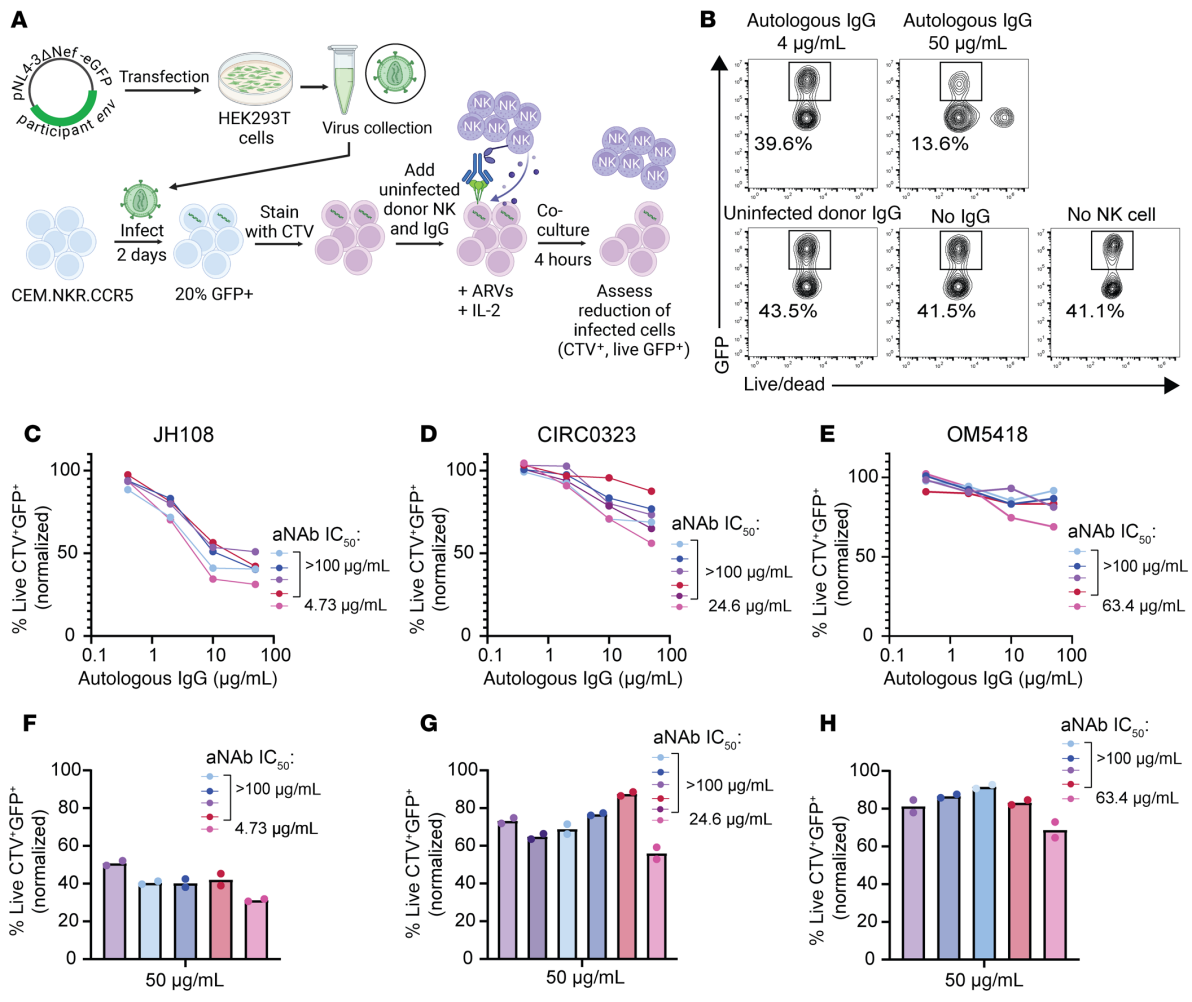
Cells infected with outgrowth viruses are susceptible to ADCC with autologous IgG obtained after long-term ART. (**A**) In vitro ADCC assay. Participant-specific *env* genes from outgrowth viruses were cloned into NL4-3-ΔNef-eGFP backbone to generate replication-competent reporter viruses. Target CEM.NKR.CCR5 cells (88) were infected with reporter viruses and after 2 days were stained with Cell Trace Violet (CTV). Target cells were cocultured with uninfected donor NK cells and autologous IgG in the presence of antiretroviral drugs (ARVs) and IL-2 for 4 hours. The reduction of infected cells was assessed by flow cytometry. (**B**) Representative flow cytometry plots showing the percentage of live GFP^+^ CTV^+^ CEM.NKR.CCR5 target cells after coculture with uninfected donor NK cells in the presence of the lowest (0.4 μg/mL) and highest concentration (50 μg/mL) of autologous IgG. Plots from control wells with uninfected donor IgG, without IgG, and without NK cells are shown. (**C**–**E**) For 3 long-term ART participants, measurements show the percentage of live GFP^+^ CTV^+^ CEM.NKR.CCR5 target cells after coculture with uninfected donor NK cells in the presence of autologous IgG. Target cells were infected with GFP-reporter outgrowth viruses (1 color each) and stained with CTV. aNAb-sensitive viruses are indicated in pink with the corresponding aNAb IC_50_ values, and aNAb-resistant viruses (IC_50_ values > 100 μg/mL) are represented in other colors. Data are reported as a mean percentage of 2 replicates normalized to uninfected donor IgG. (**F**–**H**) Data from the highest autologous IgG concentration (50 μg/mL) in **C**–**E** are replotted with the mean and 2 replicates shown. Colors correspond to the same viruses as in **C**–**E**.

We next explored whether the observed prevalence of aNAb-resistant viruses reflects a selection against cells with sensitive viruses through ADCC. Only a small subset of reservoir cells are transcriptionally active at any given time, and thus this differential killing would only become apparent over long time intervals. Therefore, we studied IgG-dependent, NK cell–mediated killing of target cells expressing autologous Envs from participants on ART for over 20 years. We generated replication-competent, NL4-3–based reporter viruses (NL4-3-ΔNef-eGFP) incorporating the *env* gene from participant-specific outgrowth viruses (Figure 6A). Next, we infected CEM.NKR.CCR5 cells (88), chosen to reduce nonspecific killing. The target cells were then incubated with NK cells purified from uninfected donors in the presence of participant IgG. Killing was measured as a reduction in live, GFP^+^ target cells (Supplemental Figure 8). We tested aNAb-sensitive and aNAb-resistant isolates from 3 long-term ART participants. Minimal nonspecific killing occurred in control cultures with uninfected donor IgG, in cultures without IgG, and in cultures without NK cells (Figure 6B). For both aNAb-sensitive and aNAb-resistant viruses, we found that autologous IgG could promote ADCC by NK cells (Figure 6, C–E). Interestingly, at the highest IgG concentration (50 μg/mL), we observed higher ADCC against target cells expressing aNAb-sensitive Envs compared with most of the corresponding participant’s aNAb-resistant Envs (Figure 6, F–H). However, there was no correlation between the neutralization IC_50_ and ADCC measured as percentage killing at 50 μg/mL IgG (not shown). The mixture of neutralizing and nonneutralizing antibodies in purified IgG samples complicates analysis of the effect of neutralizing antibodies on ADCC, and multiple studies highlight the role of nonneutralizing antibodies in ADCC (89–92). Nevertheless, our results suggest that the ADCC activity of aNAbs may play a role in selection, providing one explanation for the high resistance to aNAbs after 20 years of ART.

### Large aNAb-resistant outgrowth clones remain stable in size.

**Figure 7:**
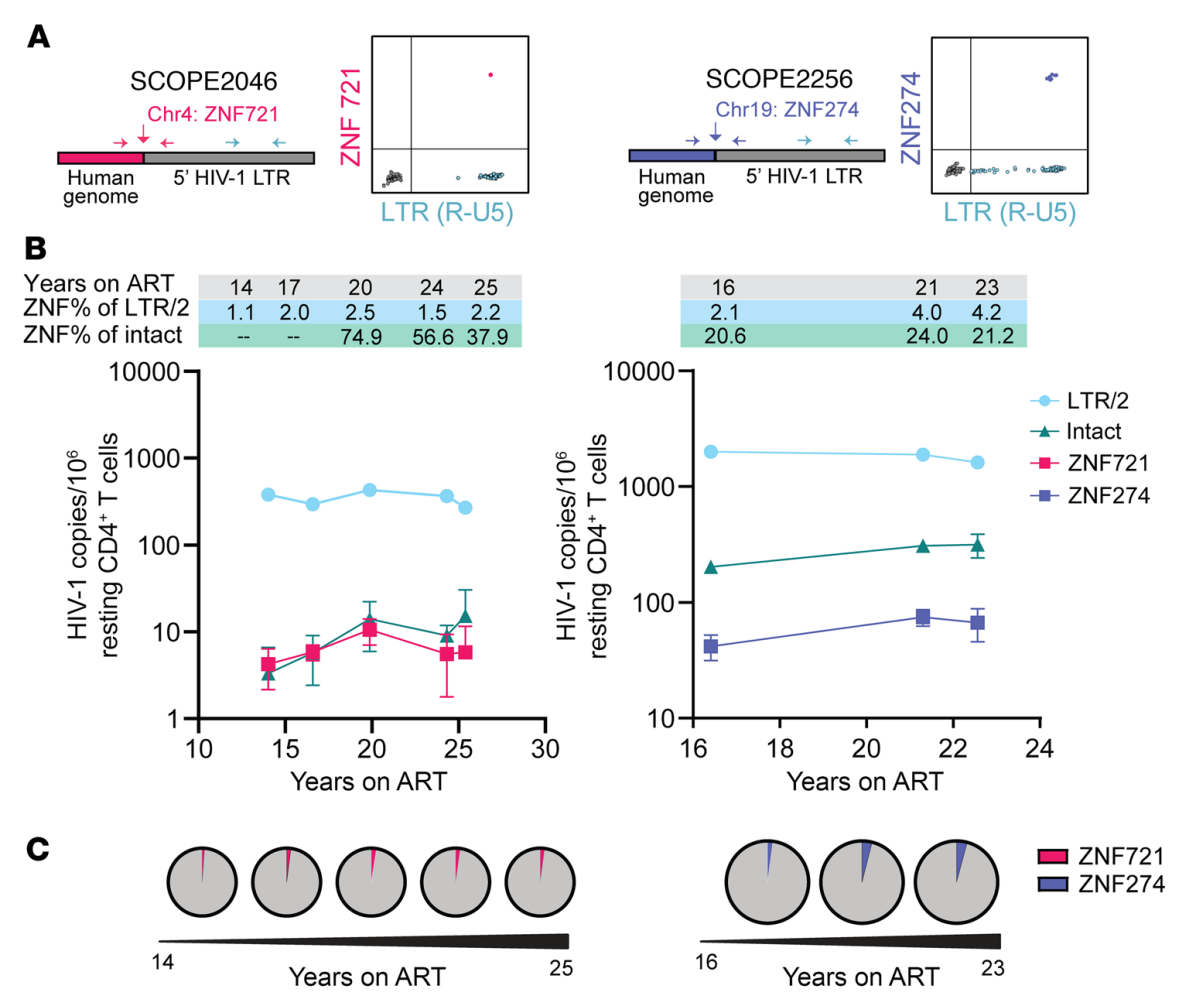
Persistence and prevalence of aNAb-resistant outgrowth clones integrated in ZNF regions. (**A**) Duplex digital droplet PCR (ddPCR) assays were designed to capture the frequency of proviruses with a specific integration site and *env* sequence matching an aNAb-resistant outgrowth clone. ddPCR plots display representative data from one well. (**B**) Longitudinal quantification of the SCOPE2046 ZNF721 and SCOPE2256 ZNF274 proviruses over time on ART. ZNF frequencies (pink or purple) and LTR/2 frequencies (blue) were determined by custom ddPCR assay. Intact proviral frequencies (green) were determined by IPDA (101). Data from 2–16 replicates are shown as proviral copies per 1 × 10^6^ resting CD4^+^ T cells (mean, SEM) after correction for DNA shearing. ZNF percentages of LTR/2 and intact proviruses are shown above the graph at the corresponding time on ART. Dashes (--) represent ZNF frequencies larger than the intact proviral frequency. (**C**) Pie charts depicting proportion of ZNF721 (pink) or ZNF274 (purple) out of respective detected LTR/2 copies for each time point.

Given the potential role of aNAbs in selecting against cells carrying sensitive variants, we hypothesized that large clones of infected cells carrying aNAb-resistant viruses might persist in PWH on very long-term ART. To definitively identify infected cell clones, we used matched integration site and near full-length proviral sequencing (MIP-seq) (93). Integration sites were matched to the *env* sequences of 2 large clones, each from a different PWH on long-term ART in the aNAb-resistant group (Figure 7A). For those 2 viruses, we designed a duplex digital droplet PCR (ddPCR) assay using primers and probes spanning the virus-host junction (integration site) as well as the HIV-1 LTR (R-U5), to determine the frequency of the clones among resting CD4^+^ T cells (94). One clone from participant SCOPE2046 gave rise to 11 QVOA isolates (Supplemental Figure 2) and was resistant to aNAbs (IC_50_ of approximately100 μg/mL) from both timepoints tested, after 4.5 and 25.3 years on ART (Figure 5F). The integration site was chr4: 451609(+), within the ZNF721 gene. ZNF genes have been associated with regions of heterochromatin, leading possibly to deeper proviral latency (95, 96). Large clones integrated into ZNF721 have been previously detected in PWH on ART and elite controllers, in some cases with viral outgrowth assays (69, 93, 97–99). Duplex ddPCR analysis on 5 cell samples spanning 11 years of ART showed that the frequency of this clone was stable (average 6.4/million resting CD4^+^ T cells; Figure 7, B and C). This was 1.1%–2.5% of the average frequency of all proviruses assayed (347 of 1,000,000 resting CD4^+^ T cells, determined as half of the LTR frequency). For perspective, approximately 90% of proviruses are defective in PWH on ART (100). The average frequency of intact proviruses in this participant was 9.5 copies/million resting CD4^+^ T cells, as determined by the IPDA (101), using the same cell samples. Thus, on average, this stable resistant outgrowth clone makes up 67% of all intact proviruses, consistent with the highly aNAb-resistant reservoir in this donor (Figure 1B). The second integration site match was for the major outgrowth clone (23 isolates) from SCOPE2256 (Supplemental Figure 2). This clone showed aNAb resistance (IC_50_ > 100 μg/mL) over 13 years of ART (Figure 5G). The integration site was chr19: 58193728(–), within the ZNF274 gene. Chromosome 19 is enriched with repressive chromatin marks covering ZNF genes (95, 102, 103). Proviral clones integrated in chromosome 19 (19, 69), including ZNF274 (104), have been identified in elite controllers and PWH on ART. In 3 samples spanning 6 years of ART, the frequency of this provirus remained stable (average 61.2/million resting CD4^+^ T cells; Figure 7, B and C). The average frequency of total proviruses was 1,835 copies/million resting CD4^+^ T cells, and the average frequency of intact proviruses was 277.2/million resting CD4^+^ T cells. Thus, this resistant outgrowth clone is a small fraction of all persistent proviruses (2.1%–4.2%) but is a substantial proportion of the intact reservoir, on average nearly 25%. These data support long-term persistence of large aNAb-resistant outgrowth clones that are inducible in the QVOA despite integration into ZNF genes and are potentially capable of causing rebound due to aNAb resistance.

## Discussion

aNAbs block outgrowth of a fraction of inducible, replication-competent reservoir viruses (30, 105). Here, we analyzed aNAb activity against outgrowth viruses from 31 PWH on ART. Substantial aNAb resistance (IC_50_ of contemporaneous IgG: > 100 μg/mL) was found in the majority of participants using multiple analyses: fraction of all distinct isolates, fraction of all isolates including members of a potential clone, and those parameters on a per person basis. For downstream analysis, these participants were split into groups with high or low aNAb resistance. Resistance to neutralization tended to correlate with longer times on uninterrupted ART.

Using a previously described pharmacodynamic metric (IIP) for logs of inhibition produced by an antiviral agent (80–82, 84), we predicted the in vivo activity of aNAbs at physiologic IgG concentrations, which are much higher than antibody concentrations used in neutralization assays. This analysis revealed that many isolates are actually susceptible to neutralization, but with IIP values of 1–3 (1–3 logs of inhibition of single round infection at physiologic IgG concentrations). This is in the range observed for many single antiretroviral drugs (80, 82, 85), a level of inhibition that leads to rapid selection of resistance. Previous studies have shown that IIP values greater than 5 are required to produce complete suppression of viral replication (80, 82, 85). This level of inhibition has been observed in a recent case of posttreatment control that appears to be mediated by aNAbs (106).

We found that only rare isolates were neutralized at this level. Importantly, although few replication-competent isolates are potently neutralized by aNAbs in in vitro neutralization assays, a lower degree of neutralizing activity may be sufficient to prevent viral rebound from individual latently infected cells once suppression of viral replication has been achieved.

A consistent feature of effective neutralization is a high slope or Hill coefficient, reflecting cooperativity in the action of the antibody (107). Previous studies of bNAbs showed that antibodies targeting different regions of the Env trimer have different slopes (83). The highest slope values, approximately 1.5, were observed for antibodies targeting the CD4 binding site and the V3 glycan epitopes. Regarding mechanism, some classes of antiretroviral drugs have cooperative dose-response curves even though the drugs target viral proteins that are univalent with respect to the inhibitor (107). This form of cooperativity is observed when infectivity rather than enzyme activity or binding is the readout and multiple copies of the targeted viral protein participate in the relevant step in the virus life cycle. For antibodies, additional factors may give rise to cooperative dose-response curves, including the bivalent nature of IgG, the trimeric nature of the Env spike, the possible requirement for multiple trimers to mediate fusion, and, for aNAbs, the cooperation between noncompeting antibodies that bind distinct epitopes. For example, it is possible that binding of one IgG induces a conformational change that facilitates the binding of additional antibodies.

The observed resistance to aNAbs was not solely due to general waning of the antibody response. Antibodies from participants generally neutralized the tier 1 virus SF162 (86, 87). Although longitudinal samples spanning multiple decades were only available for 3 participants, we showed that for these three PWH, the ability to neutralize SF162 was maintained over 13–21 years of ART. Over the same time intervals, we observed waning, stability, and improvement of aNAb activity against autologous outgrowth viruses, depending on the participant. These results suggest that some aNAbs can persist over 20 years of suppressive ART. Other studies have shown that aNAb responses can mature and persist in the presence of ART (74, 108–111).

To investigate other mechanisms for increased aNAb resistance, we conducted in vitro ADCC assays with target cells expressing participant-derived *env* sequences. For 3 long-term ART participants, we found ADCC activity against cells expressing all of the relevant donor Envs, with a preference for killing of cells expressing aNAb-sensitive Envs. However, purified autologous IgG contains both neutralizing and nonneutralizing antibodies to Env at unknown proportions (89–92, 112). This makes it difficult to distinguish the degree of selection mediated by the 2 types of antibodies. Many antibodies to the Env protein are not neutralizing, binding instead to epitopes exposed on open Env trimers, monomeric gp120, or gp41 stumps (113). Nonneutralizing antibodies have been shown to have a protective effect, exerting partial control of viremia early during infection (114). Both types of antibodies can select for escape mutations (92, 115). Nonneutralizing antibodies appear within 2–4 weeks of infection (116). They can mediate multiple Fc-mediated effector functions, including the release of inflammatory mediators, phagocytosis, and ADCC (117). In the RV144 vaccine trial, the only human vaccine trial to demonstrate measurable efficacy, higher ADCC activity was associated with protection (118, 119). Yet, in the follow up trial HVTN702, no efficacy was demonstrated (120).

Our study has limitations. Because longitudinal samples spanning over 2 decades of suppressive ART are generally not available, we compared differences in aNAb activity cross-sectionally among PWH who had been on ART for different lengths of time. Nevertheless, the observed aNAb resistance in PWH on very long-term ART was not dependent on the comparison cohort. Definitive conclusions regarding the mechanism of resistance will require longitudinal analysis of samples collected over multiple decades on ART. A complication with the ADCC assay is that these experiments used NK cells from uninfected donors, rather than matched donor cells. During HIV-1 infection, NK cells undergo progressive dysregulation (121), which may impact the efficiency of ADCC-mediated killing.

In summary, our analysis of autologous antibody function and persistence revealed a large proportion of isolates with resistance to neutralization in PWH on long-term ART. This was not exclusively due to the waning of the antibody response. For future antibody-based therapeutic approaches, it will be important to determine whether memory B cells capable of producing aNAbs can be restimulated. It is possible that the observed resistance also reflects a selection process that favors aNAb-resistant viruses. These results emphasize the importance of starting ART early to limit the size of the reservoir (122) and the evolution of aNAb-resistant escape variants.

## Methods

### Sex as a biological variable.

Sex was not considered as a biological variable. Both sexes were represented, although most participants were male. Our findings are expected to be relevant to both sexes.

### Study participants.

Participants were selected based on clinical records of viral suppression on ART (4–27 years). Acutely treated PWH (ART initiation within 2 months of infection) were excluded. All participants maintained generally undetectable plasma HIV-1 RNA levels. Five exceptions were DEL-SPC-015, -017, and -019 who previously received VRC01 and an ATI in ACTG trial A5340 (54, 61), as well as DEL-SPC-012 and JH448, who had periods of nonadherence. Peripheral blood samples or deidentified leukapheresis samples were obtained from 4 participants in the UCSF SCOPE cohort, 5 participants at the University of Toronto, 9 participants at the University of Pennsylvania, and 13 participants from the Johns Hopkins Hospital Bartlett Clinic. Participant characteristics are shown in Supplemental Figure 1 and Supplemental Table 1.

### Isolation of resting CD4^+^ T cells and plasma.

Peripheral blood mononuclear cells (PBMCs) and plasma were separated from blood and leukapheresis samples by Ficoll gradient centrifugation. Plasma was stored at –80°C. Total CD4^+^ T cells were isolated using the EasySep Human CD4^+^ T Cell Enrichment Kit (19052, STEMCELL Technologies). Resting CD4^+^ T cells were isolated from CD4^+^ T cells by negative selection via CD25 Microbeads II, CD69 MicroBead Kit II, and anti-HLA-DR Microbeads (130-092-983, 130-092-355, 130-046-101, Miltenyi Biotec).

### Quantitative viral outgrowth assay.

The quantitative viral outgrowth assay QVOA was set up with purified resting CD4^+^ T cells as previously described (15, 77, 78). After day 21, an HIV p24 ELISA assay (NEK050A001KT, Revvity) was carried out on culture supernatants. Infectious units per million (IUPM) resting CD4^+^ T cells were determined using maximal-likelihood limiting-dilution statistics with IUPMStats v1.0 as previously described (123). p24^+^ supernatants were stored at –80°C.

### Viral RNA isolation, cDNA synthesis, and sequencing of env.

Thawed p24^+^ QVOA supernatants were spun at 5,000*g* at 4°C for 15 minutes to remove cells and debris and then RNA was extracted using 96-well spin plates according to the manufacturer protocol (Zymo Research). Eluted RNA was reverse transcribed with SuperScript III Reverse Transcriptase (Invitrogen) using the envelope-specific primer env3out (5′-TTGCTACTTGTGATTGCTCCATGT-3′) (124) as previously described (15). The cDNA was used as a template for full-length *env* sequencing by nested inner and outer PCRs, as described previously (124), with the modified primer env3in (5′-TTTGACCACTTGCCACCCAT-3′) (15). Input DNA was run at limiting dilution so that less than 30% of wells were positive across 12 replicates after visualizing on 1% agarose gels. PCR products (~3kb) were then cleaned using the Monarch PCR and DNA Cleanup Kit (New England Biolabs) according to the manufacturer protocol and submitted for Sanger sequencing (Azenta) using the primers env5out (124), env3out (124), BKRev16 (22, 30), and BKFor16 (22, 30).

### Isolation of autologous IgG.

Plasma was heat inactivated at 56°C for 40 minutes. Autologous IgG antibodies were purified using NAb Protein A Plus Spin Columns (Thermo Fisher Scientific) according to the manufacturer’s protocol. Buffer was exchanged multiple times using Slide-A-Lyzer G3 Dialysis Cassettes, 10K MWCO (Thermo Fisher Scientific) in phosphate-buffered saline (PBS), pH 7.2, at 4°C. IgG was collected and sterile-filtered in 0.22 μM centrifugal filter tubes (Millipore). IgG concentrations were determined by absorbance at 280 nm on a microvolume UV-Vis spectrophotometer (Thermo Fisher).

### HIV-1 Western blot.

Qualitative detection of HIV-1 antibodies was performed using the GS HIV-1 Western Blot Kit (32508, Bio-Rad) according to the manufacturer’s protocol.

### Single-round pseudovirus generation.

For neutralization assays, full-length HIV-1 *env* sequences were transferred into *env* expression vectors by TA cloning. Additional 3′ A overhangs were first added by incubation with 0.2 mM dNTPs (Thermo Fisher Scientific), 1x PCR Buffer providing 1.5 mM MgCl (Qiagen), and 1U Taq DNA Polymerase (Qiagen) in a 20 μl reaction for 20 minutes at 72°C. After purification with the Monarch PCR and DNA Cleanup Kit (New England Biolabs), the PCR products were cloned using the pcDNA 3.4 TOPO TA Cloning Kit (Thermo Fisher Scientific). Plasmids were screened by colony PCR using Quick-Load Taq 2X Master Mix (New England Biolabs) and Sanger sequencing (Azenta). Colonies with HIV-1 *env* in the correct orientation were purified by ZymoPURE II Plasmid Maxiprep Kit (Zymo Research). Then, 12.5 μg of the purified plasmid were cotransfected with 15 μg of pNL4-3-ΔEnv-GFP using Lipofectamine 3000 Transfection Reagent (Thermo Fisher Scientific) into HEK293T cells (ATCC) in the presence of 2.5 μg of pAdvantage (Promega) to enhance protein expression. The SF162 *env* was provided by Michael S. Seaman. Viral supernatants containing isolate-specific Env-expressing pseudoviruses were harvested 65 hours after transfection, centrifuged to remove debris, filtered with 0.45 μM centrifugal filter tubes (Millipore), snap frozen on dry ice, and stored at –80°C.

### Neutralization assays.

Pseudoviruses were titrated on TZM-bl cells (125, 126) (NIH AIDS Reagent Program) to determine the linear range for infection as described previously (126). HIV-1 Env pseudoviruses were added to serially diluted IgG and preincubated for 90 minutes at 37°C. Then, 10,000 TZM-bl cells were added with a final concentration of 50 μg/mL DEAE-dextran. Triplicate control wells were set up with cells only (no virus, no antibody) and cells with virus (no antibody). After 48 hours at 37°C, infection was measured by quantification of luciferase using the Bright-Glo Luciferase Assay System (Promega), according to manufacturer instructions, and luminescence detection on a Varioskan LUX Multimode Microplate Reader (Thermo Fisher Scientific). IgG dilutions that inhibited 50% of viral infection (IC_50_) were determined as previously reported (80) using the linear portion of the median effect plot. The maximum degree of infection was determined from wells with no or low concentrations of IgG. bNAbs used in neutralization assays, VRC01 and 10-1074, were from NIH AIDS Reagent Program (ARP-12033, ARP-12477). PGDM1400 was provided by Dennis R. Burton.

### Replication-competent reporter virus generation.

For ADCC assays, participant *env* sequences were inserted into the replication-competent NL4-3-ΔNef-eGFP backbone by Gibson assembly. Participant *envs* were amplified with the forward primer: 5′-GAGCAGAAGACAGTGGCAATGA-3′ and reverse primer: 5′-gagcggccgcgccaccCATCTTATAGCAAAGCCCTTTCYAAGCC-3′ containing overhangs that match the vector backbone. PCR cycling was as follows: 98°C for 30 seconds, followed by 30 cycles of 98°C for 10 seconds, 60°C for 10 seconds, and 72°C for 2 minutes, and then a final extension of 72°C for 5 minutes. Next, the backbone was amplified with 2 reactions. The first amplification used the forward primer 5′-GATAATACCGCGCCACATAGCAGAAC-3′ and reverse primer 5′-CATTGCCACTGTCTTCTGCTCTTTC-3′. PCR cycling was as follows: 98°C for 30 seconds, followed by 30 cycles of 98°C for 10 seconds, 60°C for 10 seconds, and 72°C for 7 minutes, and then a final extension of 72°C for 7 minutes. The second amplification used the forward primer 5′-GATGGGTGGCGCGGC-3′ and reverse primer 5′-CTGCTATGTGGCGCGGTATTATCC-3′. PCR cycling was as follows: 98°C for 30 seconds, followed by 30 cycles of 98°C for 10 seconds, 60°C for 10 seconds, and 72°C for 5 minutes, and then a final extension of 72°C for 7 minutes. PCR reactions were performed using 1 ng template, 1x Superfi II Buffer, 0.2 mM dNTPs, 0.25 μM forward and reverse primers, and 2U of Platinum SuperFi II DNA Polymerase (Thermo Fisher Scientific). Reactions were digested with DpnI (New England Biolabs) by incubation for 1 hour at 37°C to remove parental plasmid DNA templates and immediately cleaned by Monarch PCR and DNA Cleanup Kit (New England Biolabs). A DNA molar ratio for the insert:backbone1:backbone2 was 2:1:1. The combined DNA was mixed with 2 μL of 5X In-Fusion Snap Assembly Master Mix (Takara Bio) in a 10 μL reaction and incubated at 50°C for 15 minutes. Then, 2.5 μL of the Gibson assembly reaction were transformed in NEB Stable Competent E. coli (New England Biolabs) and grown on LB agar plates containing 100 μg/mL ampicillin for 16 hours at 30°C. Single colonies were selected and grown in LB broth for 16 hours at 30°C. Plasmid DNA was isolated using Zyppy Plasmid Miniprep Kit (Zymo Research) and submitted for Nanopore sequencing (Plasmidsaurus). Then, 27.5 μg of the plasmid and 2.5 μg of pAdvantage (Promega) were transfected into HEK293T cells (ATCC) using Lipofectamine 3000 Transfection Reagent (Thermo Fisher Scientific). Supernatants were harvested 65 hours after transfection, centrifuged to remove debris, filtered with 0.45 μM centrifugal filter tubes (Millipore), concentrated with Lenti-X Concentrator (Takara Bio), and stored at –80°C.

### ADCC assay.

Replication-competent reporter viruses were titrated to determine the amount needed to achieve ~ 20%–30% infection of target CEM.NKR.CCR5 cells (88) (NIH HIV Reagent Program), as assessed by GFP expression. At least 20,000 target cells/condition at 10^6^ cells/mL were spinoculated with concentrated reported viruses at 1200*g* for 2 hours at 30°C. Following infection, CEM.NKR.CCR5 cells were washed and cultured for 2 days at 37°C. Uninfected donor NK cells were isolated from leukopaks (New York Blood Center) by negative selection (STEMCELL Technologies) and cultured overnight with 50 U/mL recombinant human IL-2 to maintain viability. Before coculture, infected CEM.NKR.CCR5 cells were stained with 0.5 μM CellTrace Violet (CTV, C34571 Thermo Fisher Scientific). Then, NK cells and stained infected CEM.NKR.CCR5 cells were cocultured at an effector:target ratio of 9:1 with serial dilutions of purified participant IgG and 50 U/mL IL-2 to enhance killing. Antiretroviral drugs (5 μM tenofovir disoproxil fumarate and 5 μM emtricitabine) were added to prevent new cycles of infection. After 4 hours at 37°C, cells were washed in PBS, stained with LIVE/DEAD Fixable Near-IR Dead Cell Stain Kit (1:1000 dilution, L34975 Thermo Fisher Scientific), and washed. Samples were run on an Intellicyt iQue Screener Plus. As shown in Supplemental Figure 8, data were analyzed with FlowJo by gating on the target cells (CTV^+^) and then live infected cells (live GFP^+^).

### Intact proviral DNA assay.

Genomic DNA was extracted (QIAamp DNA Mini Kit, Qiagen) from each participant’s resting CD4^+^ T cells and used in the IPDA as previously described (101).

### Integration site analysis.

Following unbiased whole genome amplification, limiting-diluted HIV-1 DNA from each well was split and separately subjected to viral sequencing and integration site analysis in MIP-Seq analysis, as described previously (93). Integration sites were obtained using integration site loop amplification (ISLA) (20).

### Duplex LTR and integration site quantification by ddPCR.

Custom ddPCR assays to quantify proviruses with ZNF721 and ZNF274 integration sites were designed based on the host-U3 junction, with the fluorescently labeled probe positioned across the integration site (28). For ZNF721, the forward primer 5′-TACCCTTTCTCCCTTCTCCA-3′, reverse primer 5′-GTTCTGCCAATCAGGGAAGTA-3′, and probe 5′HEX/TTCCCACTT/ZEN/GGAAGGGCTAGTTTAC/3′IABkFQ were used. For ZNF274, the forward primer 5′-GCACTGCATCTGGCTTATTATTT-3′, reverse primer 5′-CAATCAGGGAAGTAGCCTTGT-3′, and probe 5′HEX/TGTCAGGTG/ZEN/GTCTTTGGAAGGGATAATATAC/3′IABkFQ were used. For duplex quantification of all LTRs (R-U5) (94), the forward primer 5′-CTTAAGCCTCAATAAAGCTTGCC-3′, reverse primer 5′-GGATCTCTAGTTACCAGAGTC-3′, and probe 5′FAM/AGTAGTGTG/ZEN/TGCCCGTCTG/3′IABkFQ were incorporated into the ZNF reaction mix. Primers and probes had a final concentration of 900 nM and 250 nM, respectively. Reaction mixes contained ddPCR Supermix for probes (no dUTPs) (Bio-Rad). Parallel quantification of RPP30 was performed as described (101) to normalize for shearing and calculate cell equivalents. Genomic DNA extracted (QIAamp DNA Mini Kit, Qiagen) from resting CD4^+^ T cells was added to reactions so that droplets contained no more than one provirus. Droplets were made on the Bio-Rad Automated Droplet Generator. PCR reactions were run with the following parameters: 95°C for 10 minutes, 95°C for 30 seconds, 56°C for 2 minutes (steps 2 to 3 for 49 cycles), 98°C for 10 minutes, hold at 4°C (temperature change rate 2°C/second). Droplets were read on Bio-Rad QX200 Droplet Reader and analyzed using QuantaSoft. Frequencies of LTR and integration sites were based on shearing and cell equivalent input of RPP30.

### Sequence alignment and phylogenetic analysis.

After Sanger sequencing of *env*, contigs were assembled and checked for quality in Geneious. BioEdit was used to align sequences to HXB2 and remove defective *envs*. To identify distinct sequences, we used ElimDupes (https://www.hiv.lanl.gov/content/sequence/elimdupesv2/elimdupes.html). Maximum-likelihood phylogenetic trees were generated with PHYML 3.0 (http://www.atgc-montpellier.fr/phyml/) as previously described (30) and with bootstrapping of 100 replicates. MEGA11 (https://www.megasoftware.net/) was used to visualize the generated tree. Tree annotations were made in Adobe Illustrator.

### Statistics.

Statistical significance was calculated with normality tests, Mann-Whitney tests, Friedman test followed by Dunn’s test for multiple comparisons, and Spearman’s rank correlation using GraphPad Prism 10.4. A *P* value of 0.05 was considered significant.

### Study approval.

The Institutional Review Boards of the University of Pennsylvania (Philadelphia FIGHT), UCSF (SCOPE, Human Research Protection Program), Johns Hopkins University Bartlett Clinic, and University of Toronto (Unity Health Toronto) approved the study. All participants provided written informed consent.

### Data availability.

All HIV-1 sequences are available in the NCBI’s GenBank (accession numbers PV352008 - PV352146). Data for the figures are in the Supporting Data Values file.

## Author contributions

NFM, JV, HWSW, and MM conducted experiments. VH designed the Gibson assembly method for replication-competent reporter viruses. TST, XGY, and ML performed integration site analysis. NFM, MM, and FRS optimized and designed custom duplex ddPCR assay in Figure 7. JL, AS, KL, KM, PT, LJM, CK, RH, and SGD enrolled participants and provided participant samples with clinical histories. NFM, JV, MM, JMS, and RFS investigated the results. NFM, JMS, and RFS wrote the manuscript with feedback from all authors.

## Supplementary Material

Supplemental data

ICMJE disclosure forms

Unedited blot and gel images

Supporting data values

## Version 1 07/29/2025

In-Press Preview

## Version 2 10/01/2025

Electronic publication
