# Supplementary material for: Factors associated with resistance of HIV-1 reservoir viruses to neutralization by autologous IgG antibodies
Source: J Clin Invest. 2025 Jul 29;135(19):e194081. doi: 10.1172/JCI194081 (PMC12483561; doi:10.1172/JCI194081)
Supplement: Supplemental data [file jci-135-194081-s051.pdf]

Supplemental Material for:

Factors associated with resistance of HIV-1 reservoir viruses to neutralization by autologous IgG antibodies

Natalie F. McMyn, Joseph Varriale, Hanna W. S. Wu, Vivek Hariharan, Milica Moskovljevic, Toong Seng Tan, Jun Lai, Anushka Singhal, Kenneth Lynn, Karam Mounzer, Pablo Tebas, Luis J. Montaner, Rebecca Hoh, Xu G. Yu, Mathias Lichterfeld, Francesco R. Simonetti, Colin Kovacs, Steven G. Deeks, Janet M. Siliciano, and Robert F. Siliciano

Figures

Supplemental Figure 1. Plasma HIV-RNA levels and $CD4^+$ T cell counts of PWH on ART.
Supplemental Figure 2. Neutralization by aNAbs for reservoir viruses of PWH in the aNAb-resistant group.
Supplemental Figure 3. Neutralization by aNAbs for reservoir viruses of PWH in the aNAb-sensitive group.
Supplemental Figure 4. No correlation between aNAb IC_{50} values and the number of isolates with identical *env* sequences.
Supplemental Figure 5. Clinical history and reservoir characteristics compared between participants in the aNAb-resistant and aNAb-sensitive groups.
Supplemental Figure 6. Relationship between aNAb neutralization and time on ART.
Supplemental Figure 7. HIV-1 western blot analysis of IgG purified from plasma of PWH on long-term ART.
Supplemental Figure 8. Gating strategy for ADCC experiments illustrated using a representative control well with infected CEM.NKR.CCR5 cells and no NK cells.

Tables

Supplemental Table 1. Characteristics of study participants.
Supplemental Table 2. Isolates with IIP > 5.
Supplemental Table 3. Variable sensitivity of isolates from the aNAb-resistant group to neutralization by bNAbs.

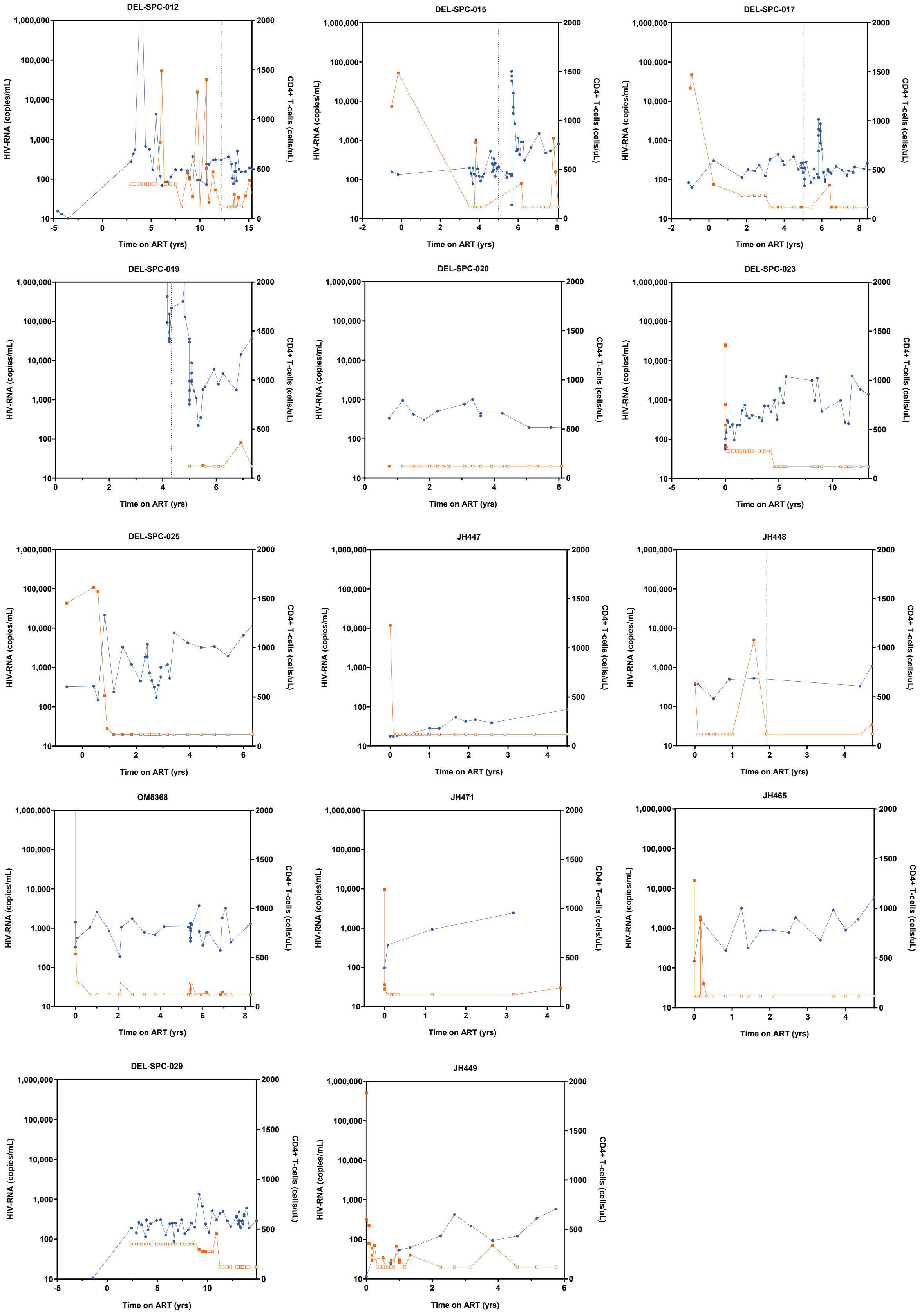
DEL-SPC-012
DEL-SPC-015
DEL-SPC-017
DEL-SPC-019
DEL-SPC-020
DEL-SPC-023
DEL-SPC-025
JH447
JH448
OM5368
JH471
JH465
DEL-SPC-029
JH449
HIV-RNA (copies/mL)
CD4+ T-cells (cells/uL)
Time on ART (yrs)

Supplemental Figure 1. Plasma HIV-RNA levels and $CD4^+$ T cell counts of PWH on ART. Levels of plasma HIV-1 RNA (copies/mL) are plotted with solid orange squares and $CD4^+$ T cells (cells/µL) are plotted in blue circles against total time on ART. Time values <0 represent measurements taken before ART initiation. Plasma HIV-RNA values below the limit of detection of the assay are indicated in open orange squares. For DEL-SPC-015, -017, and -019, viral load measurements during VRC01 administration and an ATI in the ACTG trial A5340 (IDs A02, A06, and A13, respectively) are not displayed but can be found in Bar et al. (54). For DEL-SPC-012, -015, -017, -019, and JH448, dotted vertical lines represent the starting time of uninterrupted ART.

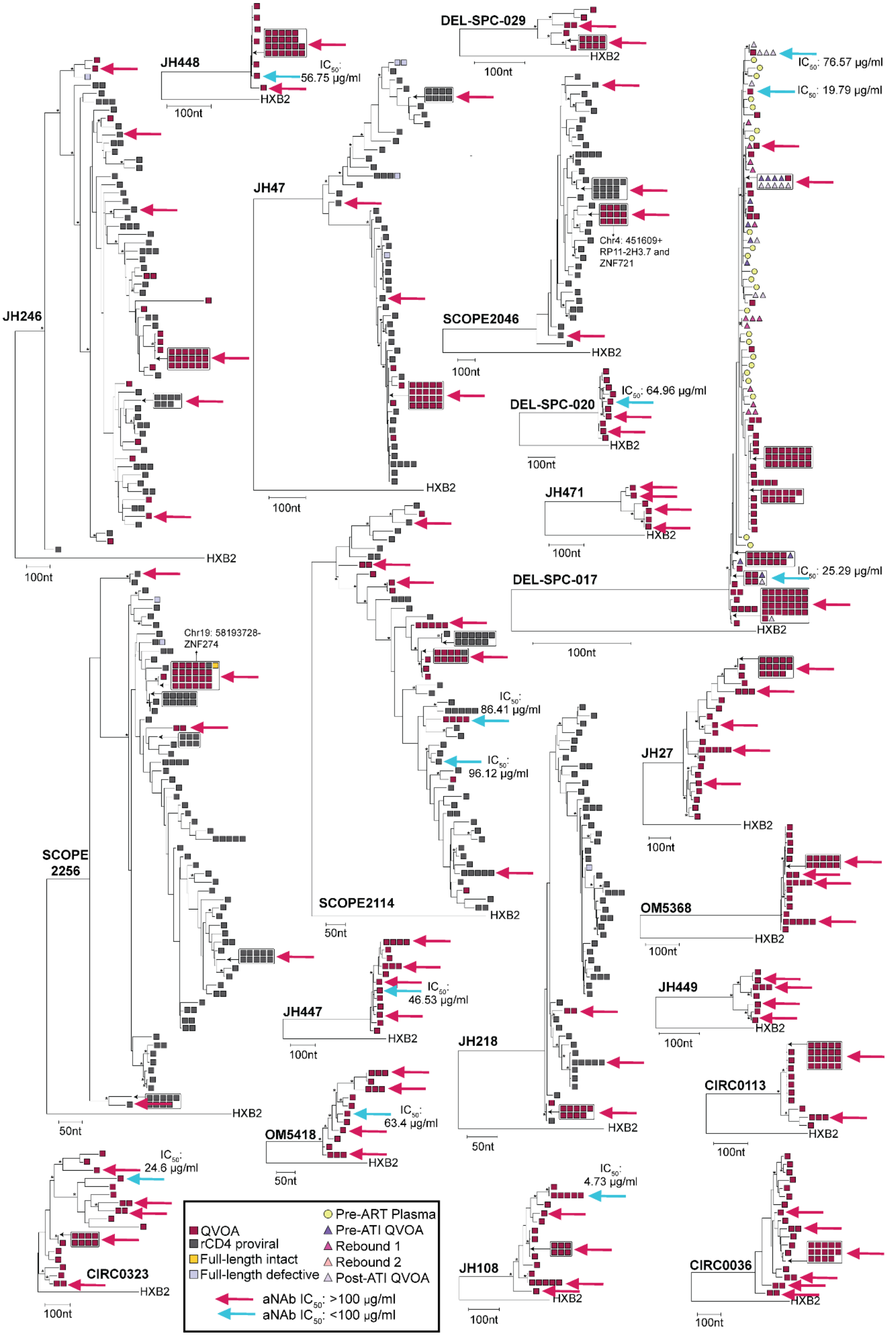
JH246
100nt
HXB2
JH448
IC50: 56.75 µg/ml
100nt
HXB2
JH47
100nt
HXB2
DEL-SPC-029
100nt
HXB2
SCOPE2046
Chr4: 451609+ RP11-2H3.7 and ZNF721
100nt
HXB2
IC50: 76.57 µg/ml
IC50: 19.79 µg/ml
DEL-SPC-020
IC50: 64.96 µg/ml
100nt
HXB2
JH471
100nt
HXB2
DEL-SPC-017
IC50: 25.29 µg/ml
100nt
HXB2
SCOPE 2256
Chr19: 58193728-ZNF274
50nt
HXB2
SCOPE2114
IC50: 86.41 µg/ml
IC50: 96.12 µg/ml
50nt
HXB2
JH27
100nt
HXB2
OM5368
100nt
HXB2
JH447
IC50: 46.53 µg/ml
100nt
HXB2
JH218
50nt
HXB2
JH449
100nt
HXB2
CIRC0113
100nt
HXB2
OM5418
IC50: 63.4 µg/ml
50nt
HXB2
CIRC0323
IC50: 24.6 µg/ml
100nt
HXB2
JH108
IC50: 4.73 µg/ml
100nt
HXB2
CIRC0036
100nt
HXB2
QVOA
rCD4 proviral
Full-length intact
Full-length defective
Pre-ART Plasma
Pre-ATI QVOA
Rebound 1
Rebound 2
Post-ATI QVOA
aNAb IC50: >100 µg/ml
aNAb IC50: <100 µg/ml

Supplemental Figure 2. Neutralization by aNAbs for reservoir viruses of PWH in the aNAb-resistant group. Maximum likelihood phylogenetic trees of intact *env* sequences for participants are shown, with each tree rooted to HXB2. Single genome sequencing of *env* was performed on cDNA reverse-transcribed from extracted viral RNA from $p24^+$ supernatants of QVOA wells (red squares) or on proviral DNA from resting $CD4^+$ T cells (rCD4, grey squares). Near full-length genome sequences were obtained from the proviral DNA and are indicated as intact (yellow squares) or defective (blue squares). For DEL-SPC-017, who previously received VRC01 and an ATI in the ACTG trial A5340 (ID A06), sequences are displayed for pre-ART plasma (yellow circles), pre- and post-ATI QVOA samples (purple and lavender triangles), and rebound virus (red and pink triangles) (54, 61). Bootstrap values greater than 70% are denoted with an asterisk. Scale units are represented in nucleotides. Red arrows indicate proviruses resistant to neutralization by contemporaneous aNAbs with IC_{50} values >100 µg/ml. Blue arrows indicate proviruses sensitive to neutralization by aNAbs with IC_{50} values at the specified value <100 µg/ml. Chromosomal integration sites obtained by integration site loop amplification and corresponding gene name are indicated. Portions of the phylogenetic trees in this figure were modified from Bertagnolli et al. (30) and McMyn et al. (15).

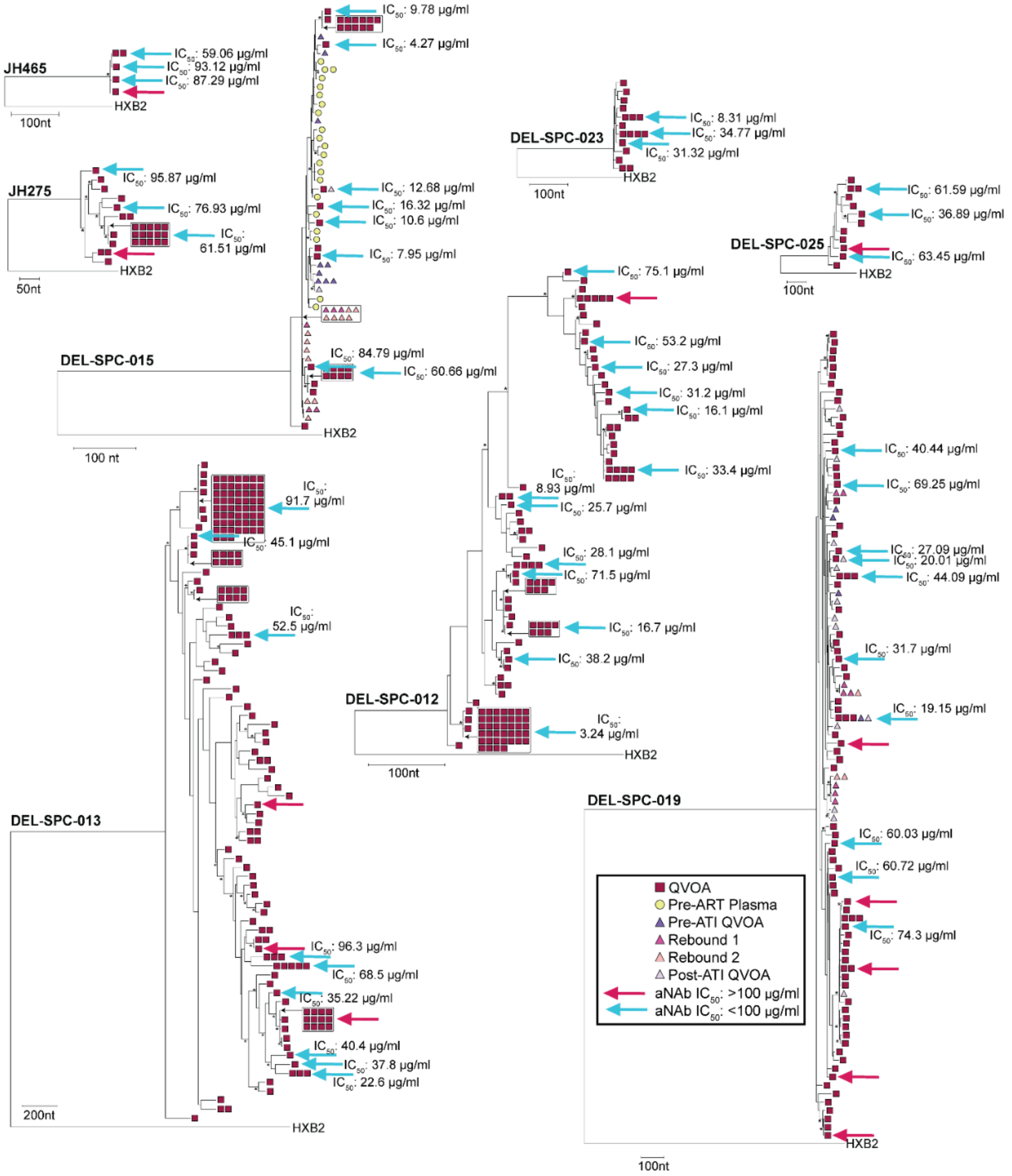

Supplemental Figure 3. Neutralization by aNAbs for reservoir viruses of PWH in the aNAb-sensitive group. Maximum likelihood phylogenetic trees of intact *env* sequences for participants are shown with each tree rooted to HXB2. Single genome sequencing of *env* was performed on cDNA reverse-transcribed from extracted viral RNA from p24^{+} supernatants of QVOA wells (red squares). For DEL-SPC-015 and -019, who previously received VRC01 and an ATI in the ACTG trial A5340 (IDs A02 and A13, respectively), sequences are displayed for pre-ART plasma virus (yellow circles), pre- and post-ATI QVOA samples (purple and lavender triangles), and rebound virus (red and pink triangles) (54, 61). Bootstrap values greater than 70% are denoted with an asterisk. Scale units are represented in nucleotides. Red arrows indicate proviruses resistant to neutralization by aNAbs with IC_{50} values >100 µg/ml. Blue arrows indicate proviruses sensitive to neutralization by aNAb with IC_{50} values at the specified value <100 µg/ml. Portions of the phylogenetic trees in this figure were modified from Bertagnolli et al. (30).

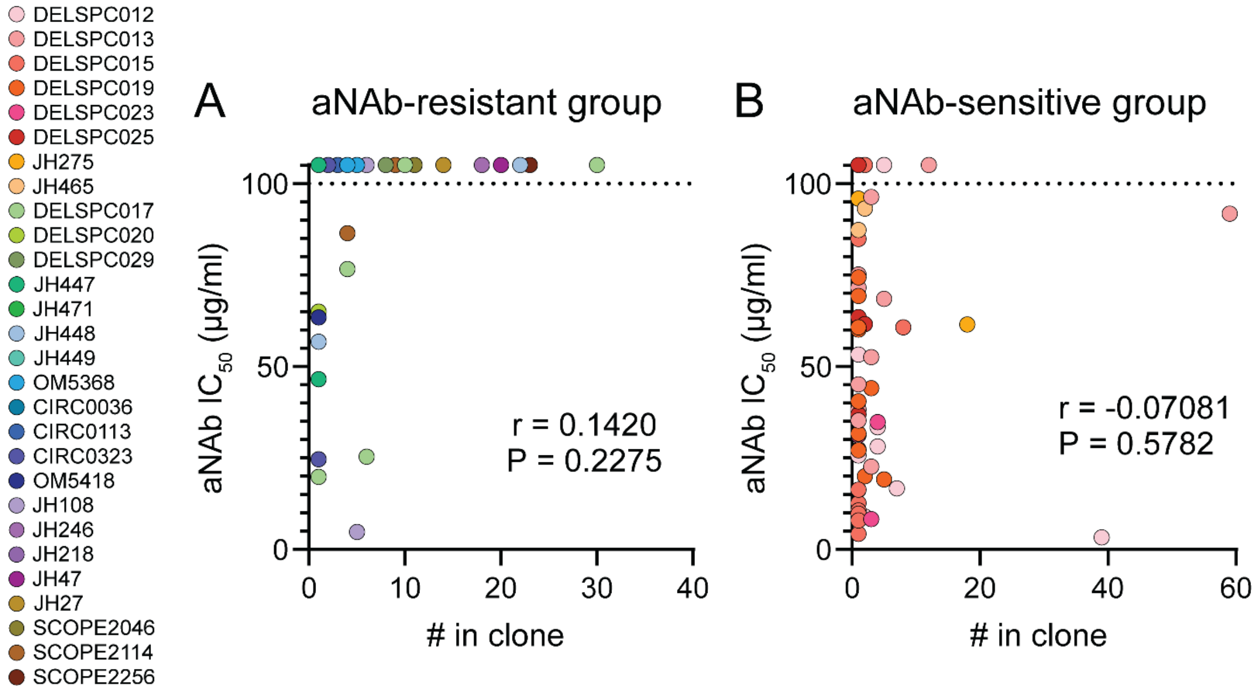

Supplemental Figure 4. No correlation between aNAb IC_{50} values and the number of isolates with identical *env* sequences. aNAb IC_{50} values for distinct isolates are replotted from Figure 2A for (**A**) the aNAb-resistant group (n = 74) and (**B**) the aNAb-sensitive group (n = 64) against the number of isolates with identical *env* sequences for the corresponding pseudovirus. Spearman's correlation values are shown.

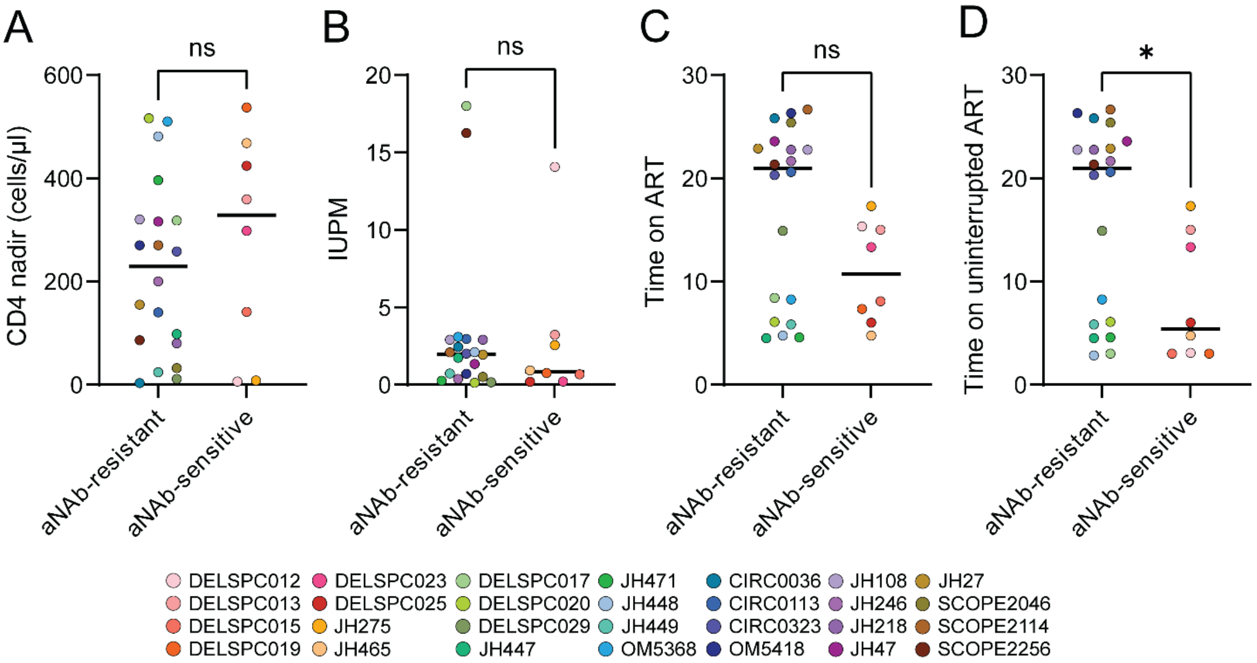

Supplemental Figure 5. Clinical history and reservoir characteristics compared between participants in the aNAb-resistant and aNAb-sensitive groups. Comparisons between aNAb-resistant group (n = 20 PWH) and aNAb-sensitive group (n = 8 PWH) values for (**A**) CD4 nadir, (**B**) IUPM, (**C**) time on ART, and (**D**) time on uninterrupted ART. Significance was calculated using the Mann Whitney test. * = P < 0.05.

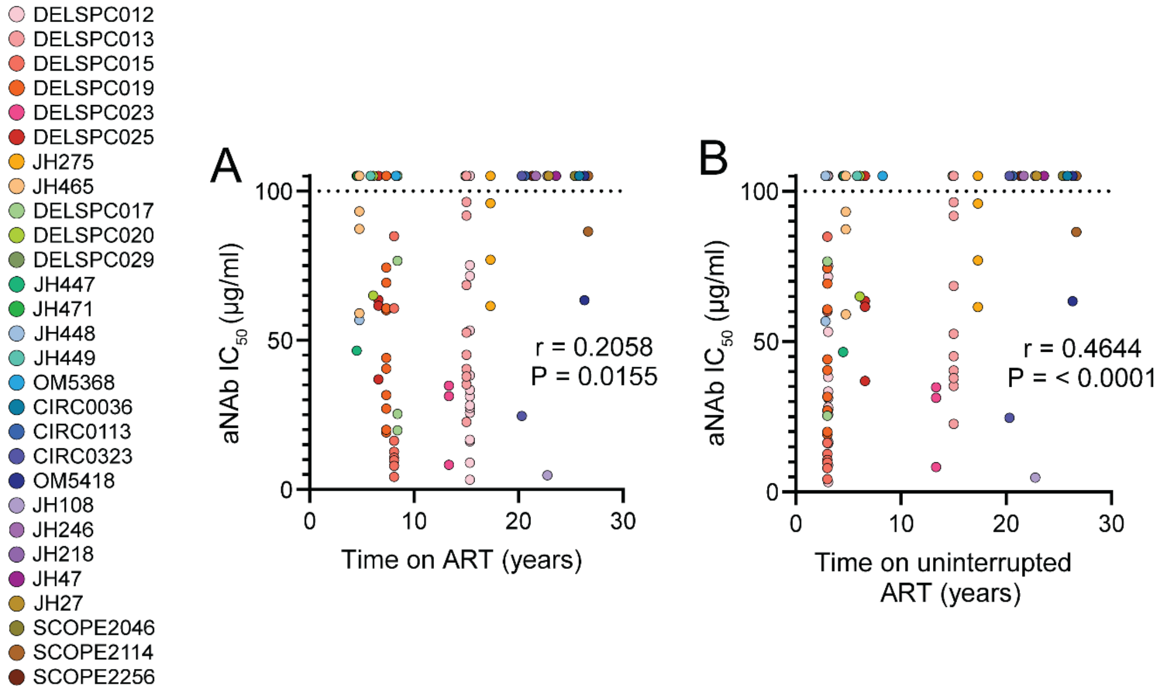

Supplemental Figure 6. Relationship between aNAb neutralization and time on ART. aNAb IC_{50} values from Figure 1A correlate with (**A**) total time on ART including short treatment interruptions (Spearman's correlation, n = 138) and (**B**) time on uninterrupted ART (Spearman's correlation, n = 138). Inclusion of multiple isolates from individual PWH give data points with the same x-axis values.

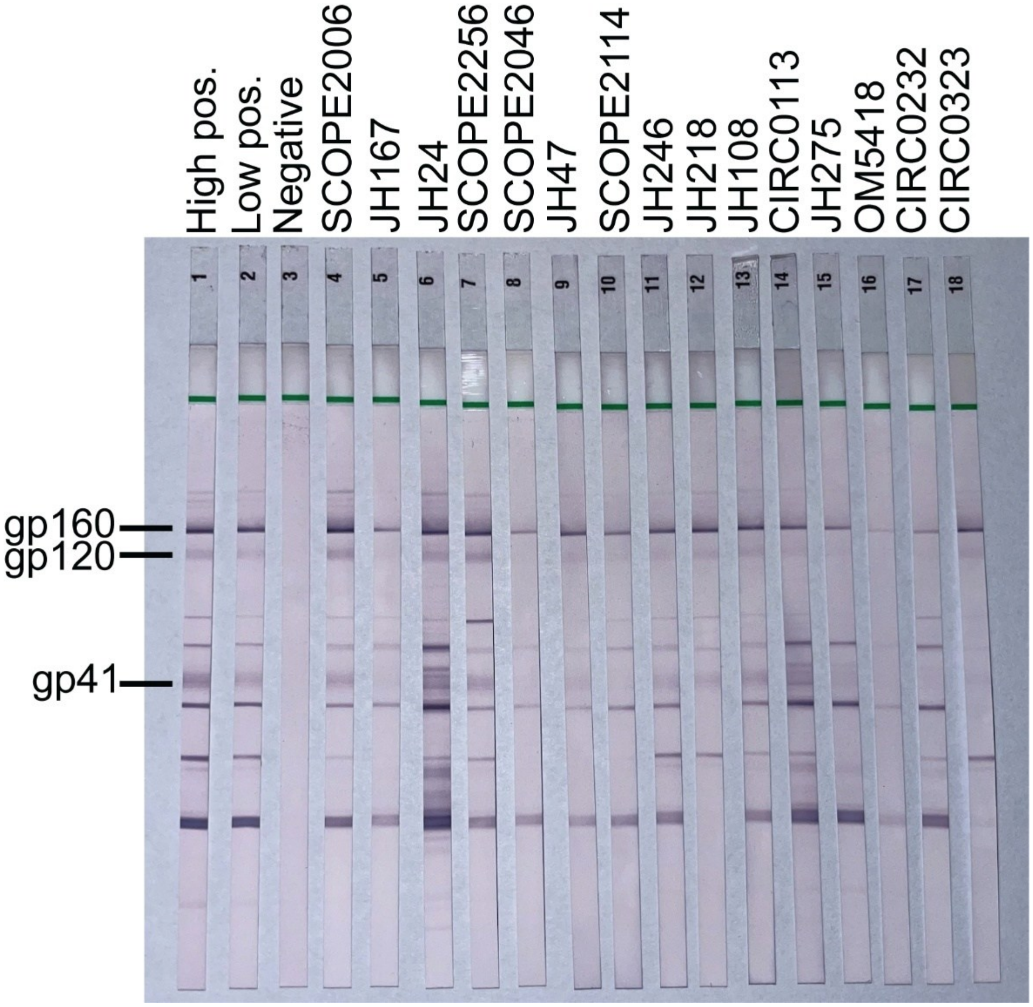

Supplemental Figure 7. HIV-1 western blot analysis of IgG purified from plasma of PWH on long-term ART. The first three columns are the high and low positive controls and the negative control. The following columns detect antibodies to HIV-1 for the indicated participants. Lanes 4-6 represent PWH with one or no outgrowth wells. Lanes 7-14, 16, and 18 represent PWH in the aNAb-resistant group. Lane 15 is a PWH in the aNAb-sensitive group. Lane 17 is a PWH on ART for over 20 years, initiating ART during acute infection and therefore excluded from this study. Bands for HIV-1 Env gp160, gp120, and gp41 are labeled.

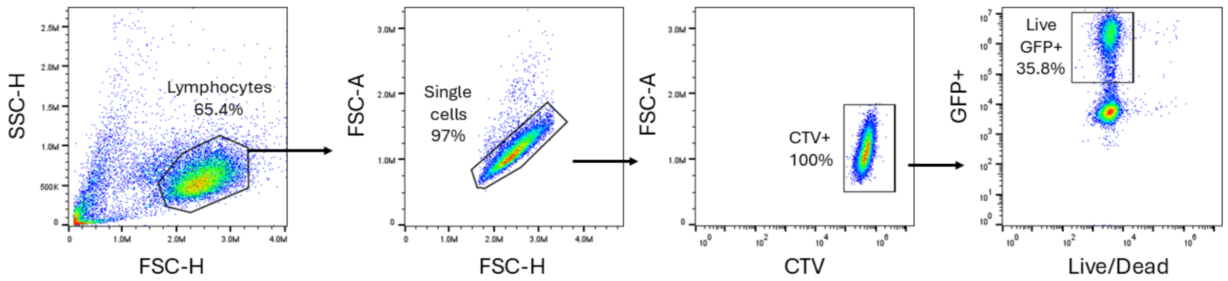

Supplemental Figure 8. Gating strategy for ADCC experiments illustrated using a representative control well with infected CEM.NKR.CCR5 cells and no NK cells. Gates were based on lymphocyte size (SSC-H, FSC-H) → single cells (FSC-A, FSC-H) → CTV^+ (FSC-A, CTV) → GFP^+, live (GFP, LIVE/DEAD Fixable Near-IR). From this analysis, we determined the percentage of CTV^+ single lymphocytes that were GFP^+ and viable: (viable,GFP^+CTV^+/CTV^+)*100. ADCC was assessed using this value normalized to the control wells with IgG from uninfected donors.

Supplemental Table 1. Characteristics of study participants.

ID	Age[A]	Sex	Race[B]	Time Since Diagnosis (Years)	Time on ART Regimen (years)	Current ART regimen[C]	CD4 nadir (cells/µl)	Plasma HIV-1 RNA (copies/mL)[A,D]
DEL-SPC-012[E]	46	M	B	21.0	15.3	ABC/DTG/3TC	6	<20
DEL-SPC-015[E]	50	M	B	8.5	8.1	ATV/r	141	<20
DEL-SPC-017[E]	57	M	B	9.5	8.4	BIC/FTC/TAF	318	<20
DEL-SPC-019[E]	51	M	B	7.4	7.3	BIC/FTC/TAF	537	<20
DEL-SPC-020[E]	34	M	B	7.1	6.1	ABC/DTG/3TC	516	<20
DEL-SPC-023[E]	51	F	B	14.6	13.3	BIC/FTC/TAF	298	<20
DEL-SPC-025[E]	42	M	B	6.6	6.0	EVG/c/FTC/TAF	424	<20
JH448	27	M	B	7.5	4.8	BIC/FTC/TAF	481	<20
JH447	32	M	B	4.5	4.5	BIC/FTC/TAF	98	<20
JH471	29	M	W	4.6	4.6	BIC/FTC/TAF	396	30
JH465	24	M	B	4.8	4.8	BIC/FTC/TAF	468	<20
JH449	35	M	B	5.8	5.8	BIC/FTC/TAF	24	<20
OM5368	60	M	W	8.3	8.3	BIC/FTC/TAF	510	<20
JH246	68	M	B	25.3	21.7	DTG/DOR	>200	<20
JH108	57	F	B	22.9	22.8	ABC/DTG/3TC	320	<20
JH47	48	M	B	30.4	23.6	BIC/FTC/TAF	316	<20
JH218	72	M	W	30.8	22.8	BIC/FTC/TAF	80	<20
JH27	68	F	B	28.8	22.9	ABC/DTG/3TC	155	<20
JH275	66	M	B	30.3	17.3	RPV/DTG	8	<20
SCOPE2046	56	M	PI	30.8	25.4	BIC/FTC/TAF, ECV	32	<30
SCOPE2256	67	M	W	36.7	21.3	DTG, RPV/TAF/FTC	86	<30
SCOPE2114	81	M	W	36.3	26.7	BIC/FTC/TAF	270	<30
CIRC0036	70	M	W	37.8	25.8	RPV/FTC/TAF/EVG/c	3	<20
OM5418	56	M	W	28.4	26.3	BIC/FTC/TAF/DOR	270	<20
CIRC0113	57	M	W	29.8	20.6	ABC/DTG/3TC	140	<40
CIRC0323	60	M	W	32.7	20.3	MVC/DOR/DTG	258	<40
DEL-SPC-013[E]	61	M	B	19.0	15.0	BIC/FTC/TAF	185	<20
DEL-SPC-029[E]	55	M	B	16.6	14.9	BIC/FTC/TAF	11	<20
JH24	62	M	B	29.8	19.1	DTG/RPV	286	<20
JH167	67	F	B	24.8	22.4	BIC/FTC/TAF	145	<20
SCOPE2006	71	M	W	27.4	25.7	FTC/TAF, DTG	9	<30
Mean	54			20.3	15.9		225	

[A]At most recent sample date.

[B]Black; W, White; H, Hispanic, PI, Pacific Islander.

[C]Abbreviations: 3TC, lamivudine; ABC, abacavir; ATV, Atazanavir; BIC, bictegravir; /c, cobicistat; DOR, doravirine; DTG, dolutegravir; ECV, entecavir; EVG, elvitegravir; FTC, emtricitabine; MVC, maraviroc; RPV, rilpivirine; /r, ritonavir; TAF, tenofovir alafenamide.

[D]Plasma HIV-1 RNA levels were measured using the Hologic Aptima and Cobas assays.

[E]Denotes participants that were included in the prior study (30).

Supplemental Table 2. Isolates with IIP > 5.

Donor_Isolate	Group	Hill coefficient[A] (*m*)	IC_{50}[A] (μg/ml)	IIP[B]	# of identical isolates	Years on uninterrupted ART
DEL-SPC-015_UD.B32	sensitive	2.20	12.68	6.37	1	3
DEL-SPC-015_UD.B18	sensitive	2.29	10.6	6.81	1	3
DEL-SPC-015_ST.A03	sensitive	1.87	9.78	5.62	1	3
JH108_A03	resistant	1.79	4.73	5.96	5	22.75

[A]Determined using the median effect equation (83).

[B]IIP of aNAbs at 10 mg/ml

Supplemental Table 3. Variable sensitivity of isolates from the aNAb-resistant group to neutralization by bNAbs.

		bNAb IC50 (μg/ml)		
Donor_Isolate	aNAb IC_{50} (μg/ml)	VRC01	10-1074	PGDM1400
JH108_A03	4.73	1.9	1.26	>4
DEL-SPC-017_ABB18	19.79	0.746	0.004	>4
CIRC0323_B46	24.6	>4	>4	0.531
DEL-SPC-017_ABB21	25.29	1.093	0.01	>4
JH447_B29	46.531	0.732	>4	>4
JH448_A07	56.75	0.172	0.114	0.726
JH5418_A14	63.4	1.27	0.038	>4
DEL-SPC-020-UDA19	64.96	1.09	>4	0.388
DEL-SPC-017_STC04	76.57	0.824	0.008	>4
SCOPE2114_B21	86.41	1.29	4	>4
DEL-SPC-017_STC11	>100	1.336	0.012	>4
DEL-SPC-017_STA01	>100	0.867	0.007	>4
DEL-SPC-017_ABB17	>100	0.731	<0.032	>4
DEL-SPC-020_STA10	>100	1.31	>4	0.323
DEL-SPC-020_STA13	>100	0.76	>4	0.39
DEL-SPC-029_ABB53	>100	>4	>4	>4
DEL-SPC-029_STB57	>100	>4	>4	>4
SCOPE2256-A15	>100	0.34	>4	>4
SCOPE2256-A24	>100	1.48	>4	>4
JH47_A07	>100	1.74	>4	0.49
SCOPE2046-A24	>100	0.75	0.53	0.16
JH246_A34	>100	1.82	0.12	>4
JH246_A22	>100	>4	0.093	0.09
JH246_A03	>100	1.45	0.61	>4
JH246_A01	>100	0.85	0.14	0.26
JH218_A09	>100	0.93	0.258	>4
JH218_A03	>100	>4	1.43	0.382
SCOPE2114_B42	>100	0.66	>4	2.19
SCOPE2114_B06	>100	1.97	>4	0.097
SCOPE2114_B01	>100	2.78	>4	0.89
SCOPE2114_B09	>100	3.42	>4	0.39

JH108_A04	>100	>4	2.2	2.1
JH108_A02	>100	3.66	0.62	>4
JH108_A14	>100	>4	2.46	0.086
JH108_A01	>100	3.45	3.31	1.57
CIRC0113_A03	>100	>4	>4	>4
CIRC0113_A09	>100	>4	>4	>4
OM5418_A03	>100	2.14	0.19	>4
OM5418_A10	>100	0.91	0.035	>4
OM5418_B39	>100	1.05	<0.032	>4
OM5418_A07	>100	0.42	0.056	>4
CIRC0323_A06	>100	>4	0.131	1.13
CIRC0323_A05	>100	2.48	0.586	1.58
CIRC0323_B03	>100	>4	>4	1.61
CIRC0323_B18	>100	0.61	>4	>4
CIRC0323_A08	>100	2	0.062	0.621
JH27_A04	>100	0.78	>4	0.5
JH27_A09	>100	0.821	>4	0.261
JH27_A11	>100	0.27	1.08	0.266
JH27_A21	>100	0.81	>4	0.22
JH27_B22	>100	1.22	2.28	0.531
CIRC0036_A13	>100	1.22	3.81	1.44
CIRC0036_A16	>100	0.44	0.74	>4
CIRC0036_B13	>100	1.67	0.79	>4
CIRC0036_B17	>100	1.79	>4	2.08
CIRC0036_B51	>100	0.74	0.48	>4
CIRC0036_C02	>100	0.74	0.73	>4
JH448_A02	>100	0.391	0.136	0.671
JH448_B07	>100	1.513	<0.032	1.008
JH447_B04	>100	0.82	>4	>4
JH447_B13	>100	0.679	>4	>4
JH447_B18	>100	0.885	>4	>4
JH447_B34	>100	0.842	>4	3.26
OM5368_A05	>100	0.111	>4	<0.032
OM5368_A07	>100	0.179	>4	<0.032
OM5368_A09	>100	0.146	>4	<0.032

OM5368_B04	>100	0.103	>4	<0.032
JH471_A07	>100	>4	2.63	<0.032
JH471_A08	>100	>4	1.55	0.127
JH471_A10	>100	>4	2.66	0.387
JH471_B08	>100	3.136	3.073	0.966
JH449_B05	>100	1.33	>4	0.106
JH449_B25	>100	2.195	>4	0.063
JH449_C02	>100	1.33	>4	<0.032

<0.032 >4 μg/ml
