# Supplementary material for: Factors associated with resistance of HIV-1 reservoir viruses to neutralization by autologous IgG antibodies
Source: J Clin Invest. 2025 Jul 29;135(19):e194081. doi: 10.1172/JCI194081 (PMC12483561; doi:10.1172/JCI194081)

Full unedited gel for Figure S7

- Figure S7 was not edited for use in the manuscript. All lanes are shown.
- Qualitative detection of HIV-1 antibodies was performed using the GS HIV-1 Western Blot Kit (Bio-Rad) according to the manufacturer's protocol.

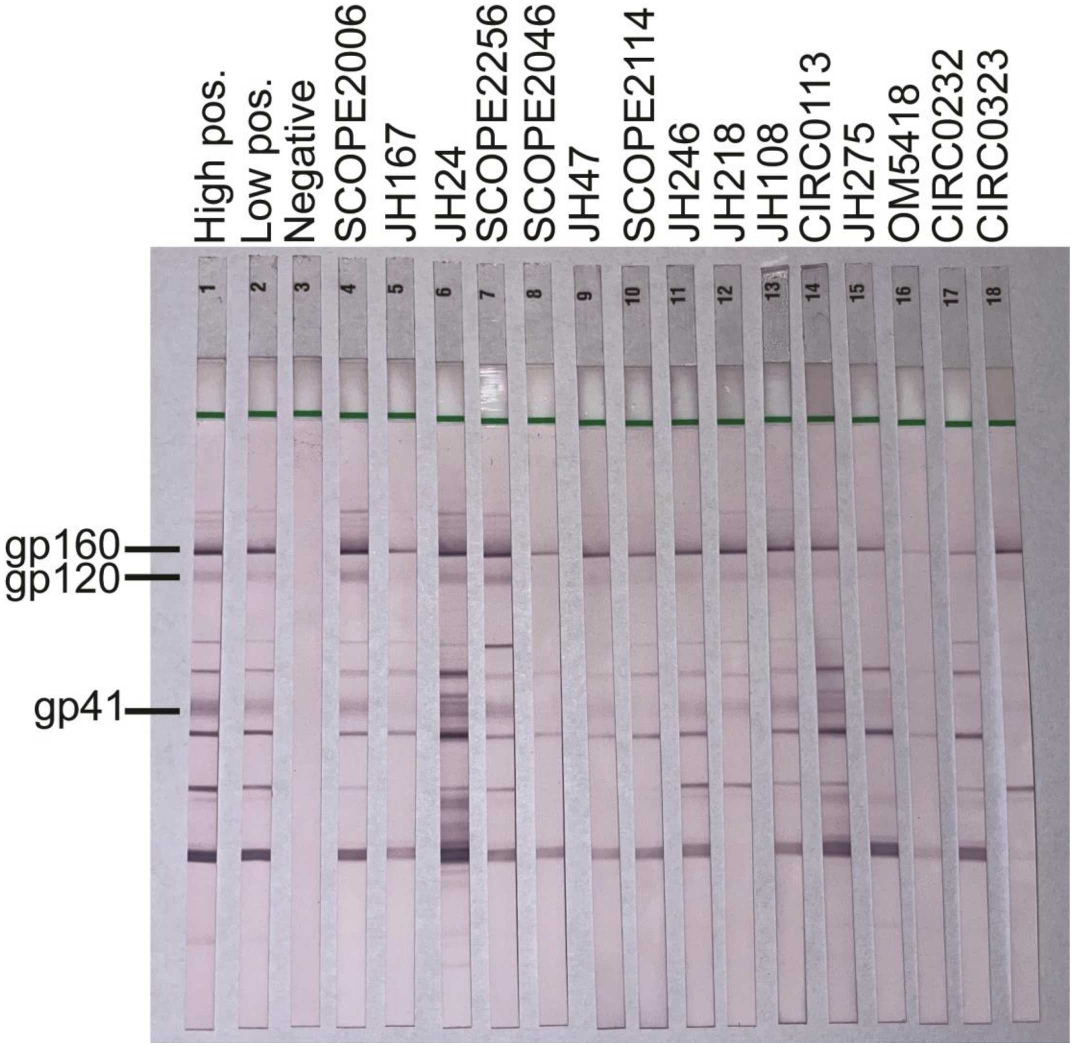

Supplement: Unedited blot and gel images [file jci-135-194081-s053.pdf]
